# Optimizing the nucleic acid screening strategy to mitigate regional outbreaks of SARS-CoV-2 Omicron variant in China: a modeling study

**DOI:** 10.1186/s40249-022-01049-w

**Published:** 2023-01-16

**Authors:** Yun Yin, Yuanhua Liu, Mengwei Duan, Xiyang Xie, Jie Hong, Jiaqi Huang, Ke Li, Jin Shi, Xi Chen, Hongyan Guo, Xuan Zhou, Rui Liu, Caifeng Zhou, Xiaozhe Wang, Lingcai Kong, Zhijie Zhang

**Affiliations:** 1 Department of Epidemiology and Health Statistics, School of Public Health, Fudan University, No.130, Dong’An Road, Xuhui District, Shanghai, 200032 China; 2 Key Laboratory of Public Health Safety, Ministry of Education, Shanghai, China; 3 Department of Mathematics and Physics, North China Electric Power University, Baoding, China; 4 Department of Blood Transfusion, Changchun People’s Hospital, Changchun, China; 5 Department of Geomatics and Spatial Information, Shandong University of Science and Technology, Qingdao, China

**Keywords:** COVID-19, SARS-CoV-2, Omicron variant, Whole-area screening strategy, Modeling

## Abstract

**Background:**

The Omicron variant of severe acute respiratory syndrome coronavirus 2 (SARS-CoV-2) spreads rapidly and insidiously. Coronavirus disease 2019 (COVID-19) screening is an important means of blocking community transmission in China, but the costs associated with testing are high. Quarantine capacity and medical resources are also threatened. Therefore, we aimed to evaluate different screening strategies to balance outbreak control and consumption of resources.

**Methods:**

A community network of 2000 people, considering the heterogeneities of household size and age structure, was generated to reflect real contact networks, and a stochastic individual-based dynamic model was used to simulate SARS-CoV-2 transmission and assess different whole-area nucleic acid screening strategies. We designed a total of 87 screening strategies with different sampling methods, frequencies of screening, and timings of screening. The performance of these strategies was comprehensively evaluated by comparing the cumulative infection rates, the number of tests, and the quarantine capacity and consumption of medical resource, which were expressed as medians (95% uncertainty intervals, 95% UIs).

**Results:**

To implement COVID-19 nucleic acid testing for all people (*Full Screening*), if the screening frequency was four times/week, the cumulative infection rate could be reduced to 13% (95% UI: 1%, 51%), the miss rate decreased to 2% (95% UI: 0%, 22%), and the quarantine and medical resource consumption was lower than higher-frequency *Full Screening* or sampling screening. When the frequency of *Full Screening* increased from five to seven times/week (which resulted in a 2581 increase in the number of tests per positive case), the cumulative infection rate was only reduced by 2%. Screening all people weekly by splitting them equally into seven batches could reduce infection rates by 73% compared to once per week, which was similar to *Full Screening* four times/week. *Full Screening* had the highest number of tests per positive case, while the miss rate, number of tests per positive case, and hotel quarantine resource consumption in *Household-based Sampling Screening* scenarios were lower than *Random Sampling Screening*. The cumulative infection rate of *Household-based Sampling Screening* or *Random Sampling Screening* seven times/week was similar to that of *Full Screening* four times/week.

**Conclusions:**

If hotel quarantine, hospital and shelter hospital capacity are seriously insufficient, to stop the spread of the virus as early as possible, high-frequency *Full Screening* would be necessary, but intermediate testing frequency may be more cost-effective in non-extreme situations. Screening in batches is recommended if the testing capacity is low. *Household-based Sampling Screening* is potentially a promising strategy to implement.

**Graphical Abstract:**

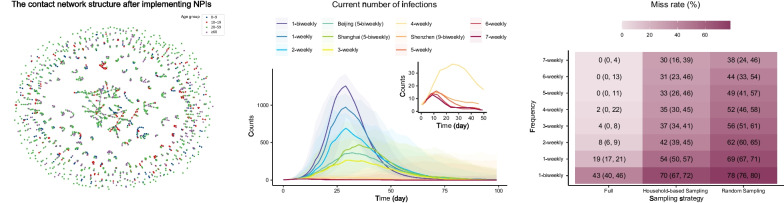

**Supplementary Information:**

The online version contains supplementary material available at 10.1186/s40249-022-01049-w.

## Background

The Omicron variant of severe acute respiratory syndrome coronavirus 2 (SARS-CoV-2), first reported in South Africa in November, 2021, rapidly became the dominant variant in the current global coronavirus disease 2019 (COVID-19) pandemic [1]. Since February, 2022, the Omicron variant has caused outbreaks in many Chinese cities, particularly in Shanghai. As of 24 May, 2022, the cumulative number of positive infections detected had exceeded 600,000, 90% of which were asymptomatic [2]. The high contagiosity of COVID-19 is led by the short incubation period and the high proportion of pre-symptomatic and asymptomatic infected persons [3–6].

The present vaccine may not effectively prevent infection caused by the Omicron variant due to its immune evasion [7]. Without non-pharmacological interventions (NPIs) such as screening and quarantine, there would be mass infections resulting in a strain on medical resources (including developed countries such as the United States and United Kingdom [8–10]). During the pandemic caused by Omicron variant, even though the mortality rate of the whole population is low, the mortality in the elderly is still high [11]. China’s extremely large population includes over 26.4 million elderly people [12]; infection of this population would lead to high demands on the health system, mass severe illnesses, and death. In the response to outbreaks, China adopted strict NPIs including mass screening, which played a key role in identifying pre-symptomatic and asymptomatic infected individuals [13, 14]. Similarly, citywide screening was carried out in Liverpool, UK, to control a COVID-19 outbreak [15]. With improvements in testing technology and the rapid establishment of testing laboratories, nucleic acid testing has become a crucial tool for controlling COVID-19 in China [16]. Screening must be performed with sufficient frequency so that latent infections in the community can be identified as soon as possible. Shanghai undertook 13 rounds of nucleic acid screenings from 13 March to 17 April, 2022, and over 200 million tests were performed [17]. However, high-frequency mass nucleic acid screening in a short period greatly increases the consumption of testing resources. The capacity of hotel quarantine is another limitation. Therefore, either overly restrictive or overly relaxed NPIs may bring tremendous public health and socioeconomic costs. Additionally, the potential risk of infection that exists when people gather and the adherence with testing strategies should be considered.

Previous studies have focused on optimizing screening strategies to reduce the time from reporting the results of testing to quarantining contacted individuals and patients [18–20], exploring optimal screening strategies in high-risk populations when testing capacity is limited [21], or evaluating the cost-effectiveness of screening strategies [22, 23]. However, it is still unclear whether a nucleic acid screening strategy can be developed that not only controls the epidemic effectively but also relieves as much as possible the consumption of resources.

In this study, we developed a stochastic network-based dynamic model that incorporates real household size, age structure, and contact patterns. It considered NPIs, such as testing, tracing, and isolation, and changes in testing sensitivity due to changes in the viral load of infected individuals over time. By simulating the spread of Omicron variant, 87 different screening strategies with different sampling methods, frequencies, and timings of screening were evaluated in terms of the cumulative infection rate, miss rate, tests per positive case identified, and the consumption of quarantine and medical resources.

## Methods

### Epidemic model

**Fig. 1:**
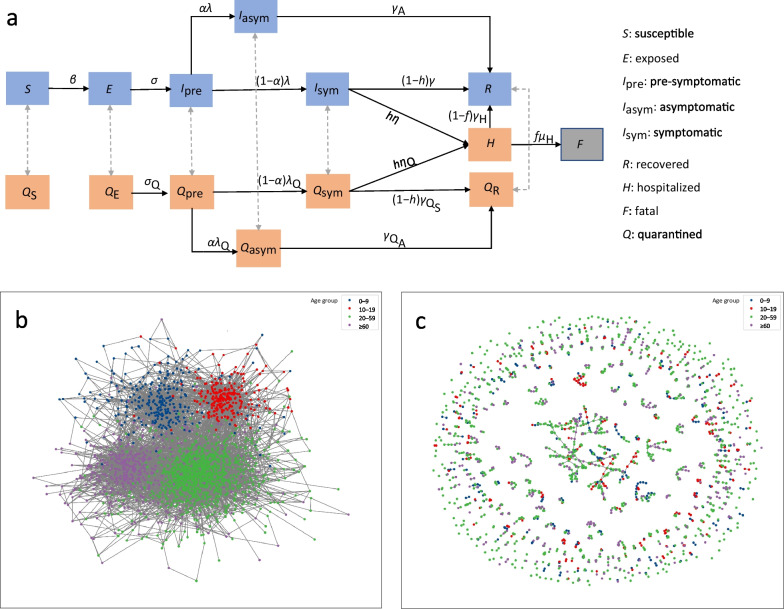
COVID-19 spread models on the network. **a** Compartmental structures. Susceptible individuals (${{S}}$) enter the latent period after being infected. After the latent period, they enter the pre-symptomatic stage, in which individuals are infectious but asymptomatic. Afterwards, some individuals develop symptoms, others remain asymptomatic, and individuals in ${{I}}_{\text{pre}}$, ${{I}}_{\text{sym}}$, and ${{I}}_{\text{asym}}$ are infectious. Some symptomatic individuals develop severe disease and are admitted to the hospital ($H$) for treatment, some of them die due to ineffective treatment ($F$), and the remaining infected individuals enter the compartment ${{R}}$ after recovery. Infected individuals who tested positive and their close contacts (which were successfully traced) enter the corresponding isolation compartment and follow the same disease course; if they do not enter other disease states after the isolation period (14 days) expires, they return to the corresponding non-isolation compartment. **b** The network structure before implementation of non-pharmacological interventions (NPIs). Individuals were represented as nodes in the network, and people of different age groups were distinguished by node color. The edges indicated the close contact between individuals, and the contact rates between individuals varied by age, individuals in the same age group were more closely connected. **c** The network structure after implementing NPIs. After the implementation of the NPIs, the individual will be isolated at home. The household members will still completely connect, but the connection with the outside of the family will be cut off

The transition of individuals between different disease processes was simulated using the Gillespie algorithm, in which the probabilities of possible events (transition between compartments) were computed, and one event was executed accordingly at each time step for a randomly selected node [24]. At each moment, individuals could be in one of the states shown in Fig. 1a, and the time spent in each state was generated randomly based on a specific probability distribution (see Additional file 1: Text S1.1 for a detailed description and Table S1 for model parameters). Considering that the current vaccines for COVID-19 only reduce the symptoms and the severity rather than prevent infection [25], we assumed that the susceptibility of individuals was homogeneous. The infectiousness of an infected individual was determined by the infection state, and the infectiousness of pre-symptomatic and asymptomatic individuals was 50% of that of symptomatic individuals [26]. Susceptible individuals can be infected through close or random casual contact (e.g., casual encounters in public) with an infectious person. We defined the proportion of contacts that were casual using the parameter ${{p}}_{\text{casual}}$ (global transmission parameter). The viral load of an individual varies over time after exposure, as does the sensitivity of nucleic acid tests. The number of days since each individual was infected was assumed to influence the sensitivity of the test. Individuals entered the corresponding quarantine compartments and followed the same disease process because of positive test results or because they were identified as close contacts. Individuals could not contact other people during the quarantine period. Therefore, once quarantined, infectious individuals do not infect others and susceptible individuals are not infected. After quarantine for 14 days, the quarantined individual returns to the non-quarantine compartment corresponding to their disease state. A proportion of the symptomatic infected cases developed severe disease requiring hospitalization, among which a small proportion die and others eventually recover. We assumed that recovered individuals are immunized and therefore cannot be reinfected.

### Network structure

We constructed a community containing 2000 people based on the age structure of the urban population and household size in 2020 [27], among which each individual was randomly assigned to different households and age groups (0–9, 10–19, … , 70–79, ≥ 80). Individuals were represented as nodes in the network, and the edges indicated close contact between individuals that was sufficient to spread SARS-CoV-2. Individuals randomly connect each other according to the contact matrices by age [28]. We assumed that all individuals within a household were fully connected and that individuals from different households were randomly connected (Fig. 1b). After the implementation of NPIs, some connections outside households were cut off; however, the household members were still completely connected (Fig. 1c). We used the parameter ${{ds}}$ (distancing scale) to describe how strictly measures were implemented.

### Testing strategies

We designed 87 whole-area nucleic acid screening strategies, considering different sampling methods, frequencies, and timings of screening (Additional file 1: Table S2). First, we designed three sampling strategies: (1) *Full Screening*, screening all people in the community; (2) *Household-based Sampling Screening*, selecting the individual with the most external connections in each family; once selected, they were screened on each screening day; and (3) *Random Sampling Screening*, randomly sampling a proportion of people in the community and then screening them on each screening day. Here, we set the sample size to be equal to the number of families. Second, we designed the frequency of screening to range between daily and biweekly. Furthermore, we also established different scenarios that screened three or four times/week. Finally, we assumed that weekly screening can be conducted in batches, which allows the individuals to be allocated into different batches and each batch is screened on different days. We also considered the Shanghai Strategy (1st, 2nd, 4th, 7th, and 14th days), Shenzhen Strategy (1st–7th, 10th, and 14th days), and Beijing Strategy (1st, 4th, 7th, 10th, and 14th days). These strategies were implemented every 2 weeks in practice.

Individuals with positive results were moved to designated hospitals or shelter hospitals for treatment according to their symptom severity, and their family members and other close contacts outside the household were moved to hotels for quarantine at the same time. Individuals who screened positive were no longer screened routinely, while others were screened according to the current screening schedule, regardless of whether they were quarantined. The screening results were associated with the individual’s infection status and infection time, we therefore assumed that the sensitivity (1 — false-negative rate) varied within 30 days after infection (Additional file 1: Table S3).

### Outcomes measured

We measured the effect of different screening strategies on the epidemic using the time series of daily number of infections, effective reproduction number (${{R}}_{\text{t}}$), cumulative infection rate (${{IR}}_{\text{cum}}$) and miss rate (1 − cumulative tested positive cases/cumulative infectors). The resources consumed in testing were measured by the number of tests performed, mean number of tests per positive case identified (cumulative number of tests/cumulative number of positive cases). The resources consumed due to quarantine, treatment in shelter hospitals, and treatment in hospitals were measured by cumulative person-days of people in hotel, shelter hospital, and hospital compartments (for example, 1 day in hotel quarantine for an individual was counted as one person-day). The peak consumption during the simulation period was also recorded. The results of each simulation were summarized by the medians and 95% uncertainty intervals (95% UIs).

### Simulations and sensitivity analysis

Initially, we assumed that all the people in the community were susceptible, except for five randomly exposed individuals. Once the infection rate reached a threshold (5/10,000 used for our simulation), NPIs would be implemented (household members are quarantined at home, but they could go out when necessary, such as purchasing necessities and seeking medical treatment). Screening with different strategies was carried out simultaneously, and the screening results were reported 1 day after the testing day. Each strategy was simulated 1000 times over of period of 100 days.

We conducted a sensitivity analysis on the basic reproduction number (${{R}}_{0}$) and proportion of asymptomatic infected ($\alpha$). We also examined different levels of NPIs by conducting sensitivity analyses on the probability of close contacts being successfully traced (${{p}}_{\text{contact}}$), global transmission parameter (${{p}}_{\text{casual}}$), and the connection between individuals and people outside the household in the contact network under the NPIs (${{ds}}$) to assess the robustness of the results. All the statistical analyses were done using Python (version 3.8; Python Software Foundation, Delaware, USA) and figures were plotted using R software (version 4.2; R Foundation for Statistical Computing, Vienna, Austria).

## Results

### Mitigating the outbreak

**Fig. 2:**
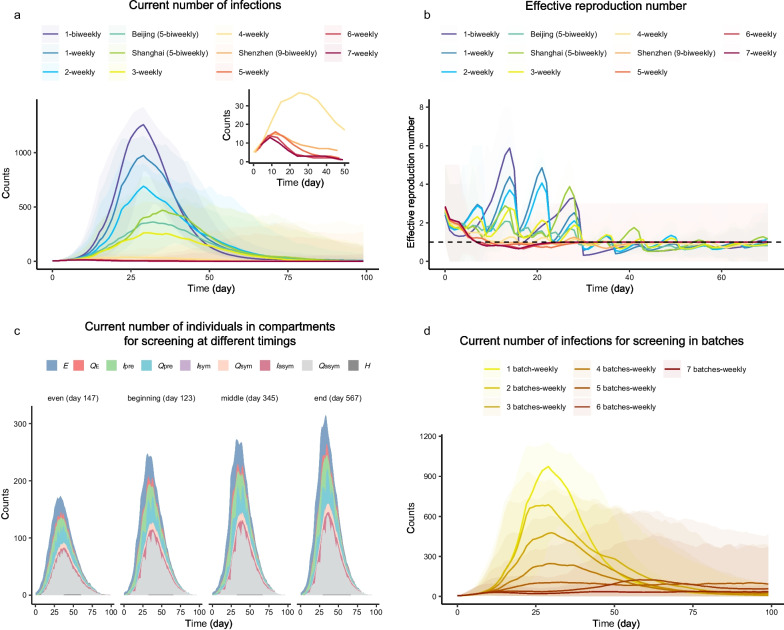
The daily number of infections of the *Full Screening* with different frequencies, timings, and batches. **a** Time series of the number of infections in various frequencies ranging from once biweekly to daily. **b** Time series of the number of effective reproduction number in various frequencies ranging from once biweekly to daily. **c** Time distribution of the number of infections in each compartment of dynamic model of screening in three times/week, which contained four screening timings in a week: evenly distributed, concentrated forward, concentrated in middle, and concentrated backward. **d** Time series of the number of infections in various batches which was from one batch to seven batches per week. The area charts represented the 95% uncertainty intervals (UIs) in **a** and **d**, and 95% confidence intervals in **b**

**Fig. 3:**
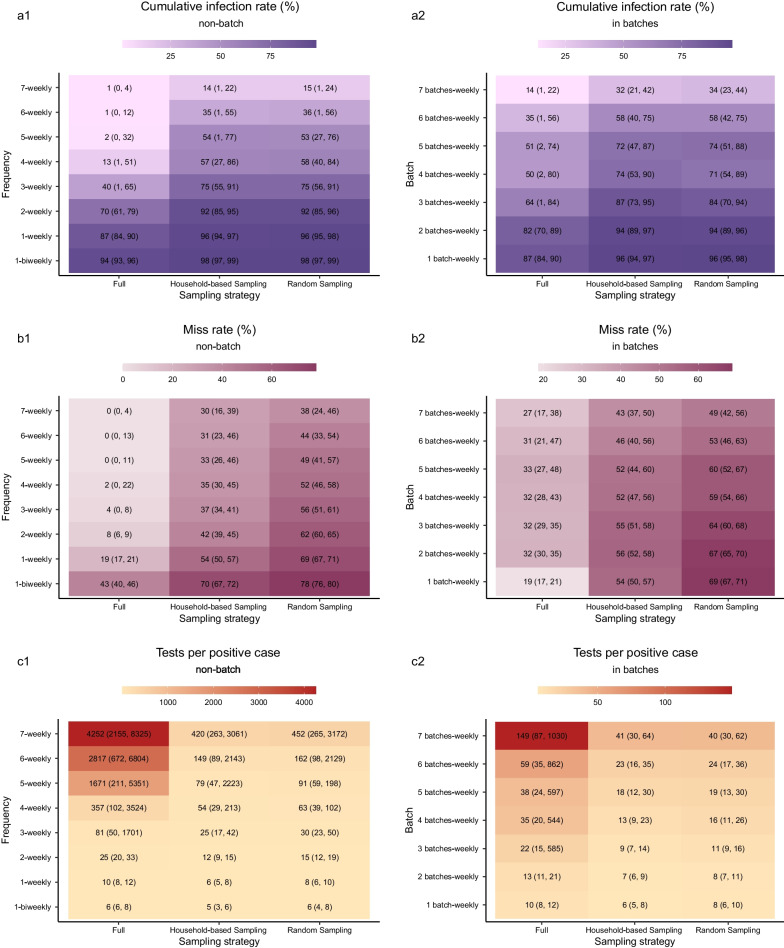
Heatmap showing cumulative infection rates, miss rates, and tests per positive case of various strategies. **a1** Heatmap of ${{IR}}_{\text{cum}}$ s at various frequencies. **a2** Heatmap of ${{IR}}_{\text{cum}}$ s in various batches. **b1** Heatmap of miss rates at various frequencies. **b2** Heatmap of miss rates in various batches. **c1** Heatmap of tests per positive case at various frequencies. **c2** Heatmap of the tests per positive case in various batches. The numbers in brackets were the 95% uncertainty intervals (UIs).

The higher the frequency of screening, the lower the cumulative infection rate (${{IR}}_{\text{cum}}$) and miss rate (Figs. 2a, 3a1, b1). If the frequency of *Full Screening* increased from biweekly to daily, the first day when ${{R}}_{\text{t}}$ < 1.00 was reduced from day 16 to day 7 (Fig. 2b), ${{IR}}_{\text{cum}}$ was reduced from 94% (95% UI: 93%, 96%) to 1% (95% UI: 0%, 4%), and the miss rate decreased from 43% (95% UI: 40%, 46%) to 0% (95% UI: 0%, 4%). What was noteworthy was that the ${{IR}}_{\text{cum}}$ decreased by 12% and the miss rate only decreased by 2%, if the frequency of *Full Screening* increased from four to seven times/week. If completing screening weekly in batches, the higher the number of batches, the lower the ${{IR}}_{\text{cum}}$ and miss rate (Figs. 2d, 3a2, b2). If batches were changed from two to seven, the ${{IR}}_{\text{cum}}$ of *Full Screening* decreased from 82% (95% UI: 70%, 89%) to 14% (95% UI: 1%, 22%), and the miss rate decreased from 32% (95% UI: 30%, 35%) to 27% (95% UI: 17%, 38%). Similar trends also occurred in *Household-based Sampling Screening* and *Random Sampling Screening*.

At the same frequency, the timing of screening affected epidemic control. For *Full Screening* as an example, although the frequency of both the Shanghai Strategy and Beijing Strategy was five times/week, the number of infections of the former was less than that of the latter in the early period, but the opposite was true in the later period (Fig. 2a). ${{IR}}_{\text{cum}}$ of the Shanghai Strategy (58%, 95% UI: 3%, 72%) was higher than that of the Beijing Strategy (49%, 95% UI: 16%, 66%). The daily number of infections during the outbreak assuming the Shenzhen Strategy (nine times every 2 weeks) was between that of screening four times/week and screening five times/week (being quite close to the latter). When the frequency was three times/week, ${{IR}}_{\text{cum}}$ and miss rate of screening with evenly distributed timing (32%, 95% UI: 1%, 57%; and 2%, 95% UI: 0%, 9%) was lower than that of screening centrally at the beginning, middle, and end of the week (Fig. 2c). As for the timings of screening in batches, ${{IR}}_{\text{cum}}$ of screening at the end of the week was higher than that of the other three options under the strict social distancing measures, although the difference between the four was not so obvious (Additional file 2: Fig. S8).

**Fig. 4:**
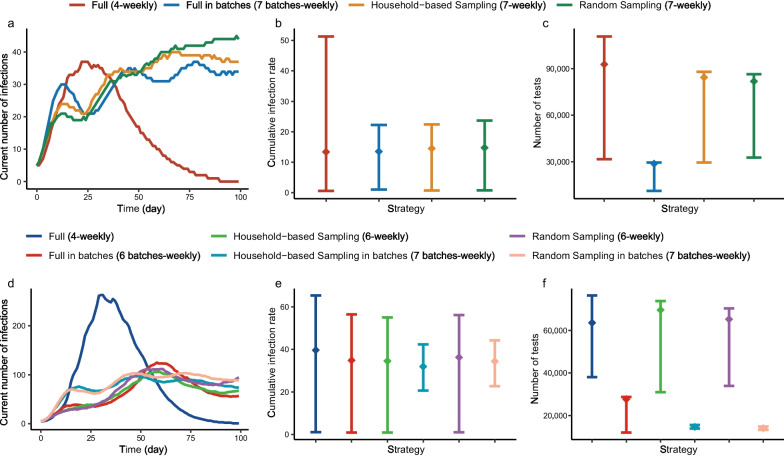
Comparison of different strategies under similar epidemic control condition. **a**–**c** Time series of the number of infections, cumulative infection rate, and number of tests in *Full Screening* four times/week, *Full Screening* in seven batches/week, *Household-based Sampling Screening* in seven batches/week respectively. **d**–**f** Time series of the number of infections, cumulative infection rate, and number of tests in *Full Screening* three times/week, *Household-based Sampling Screening* six times/week, Random Sampling six times/week, *Full Screening* in six batches/week, *Household-based Sampling Screening* in seven batches/week, Random Sampling in seven batches/week respectively. The error bars represented the 95% uncertainty intervals (UIs)

Although the two sampling strategies were not as effective in controlling the epidemic as *Full Screening*, ${{IR}}_{\text{cum}}$ of *Household-based* or *Random Sample Screening* seven times/week was similar to that of *Full Screening* four times/week (13%, 95% UI: 1%, 51%) (Fig. 4a, b). When screening in batches, ${{IR}}_{\text{cum}}$ for the *Household-based* or *Random Sampling Screening*s weekly in seven batches was similar to that for the *Full Screening* weekly in six batches (35%, 95% UI: 1%, 56%) (Fig. 4d, e). In addition, ${{IR}}_{\text{cum}}$ of *Household-based Sampling Screening* was lower than that of *Random Sampling Screening*, especially with frequency ≥ three times/week, and the miss rate of the former was obviously lower than the latter at various frequencies (Fig. 3a1, b1).

### Resource consumptions of testing, quarantining and medical treatment

With an increase in the screening frequency, the number of tests and tests per positive case identified increased (Fig. 3c1). Using *Full Screening* as an example, as the frequency increased from once biweekly to daily, the number of tests per positive case increased from 6 (95% UI: 6, 6) to 4252 (95% UI: 2155, 8325). It was notable that if screening was increased from four times/week to seven times/week, the increase in the number of tests per positive case identified was as high as 3895. When screening in batches, there was a slight increase in number of tests per positive case identified with increasing number of batches (Fig. 3c2). The tests per positive case was generally lower for *Household-based Sampling Screening* than for *Random Sampling Screening*.

**Fig. 5:**
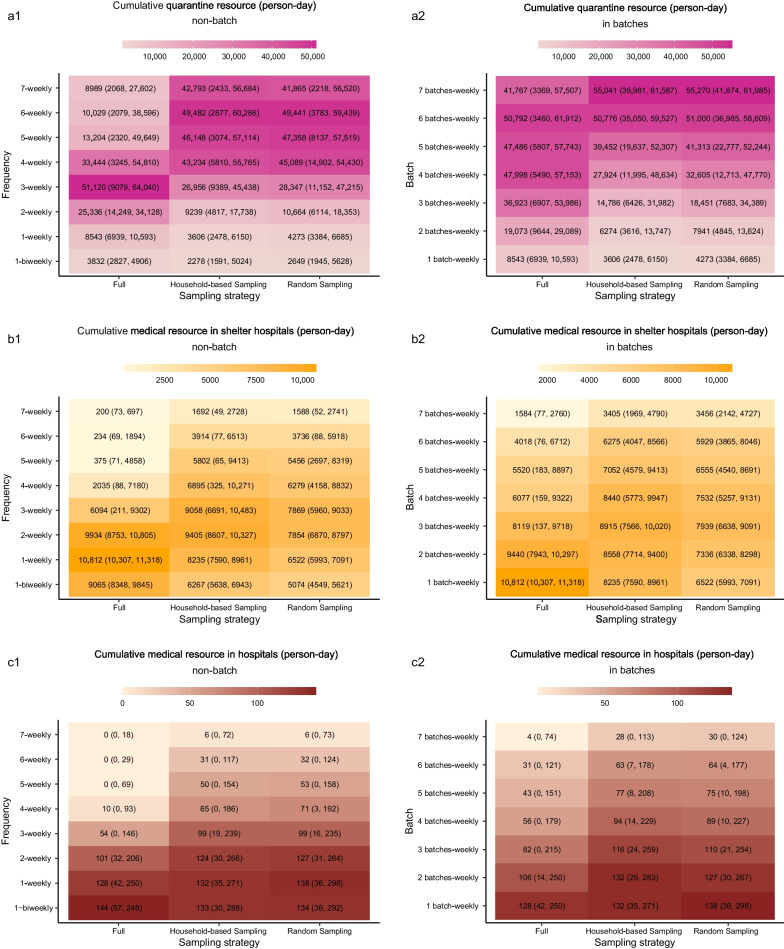
Heatmap showing the cumulative quarantine and medical resource consumption of various strategies. **a1** Heatmap of cumulative quarantine resource consumption at various frequencies. **a2** Heatmap of cumulative quarantine resource consumption in various batches. **b1** Heatmap of cumulative medical resource consumption in shelter hospitals at various frequencies. **b2** Heatmap of cumulative medical resource consumption in shelter hospitals in various batches. **c1** Heatmap of cumulative medical resource consumption in hospitals at various frequencies. **c2** Heatmap of cumulative medical resource consumption in hospitals in various batches. The numbers in brackets were the 95% uncertainty intervals (UIs)

**Fig. 6:**
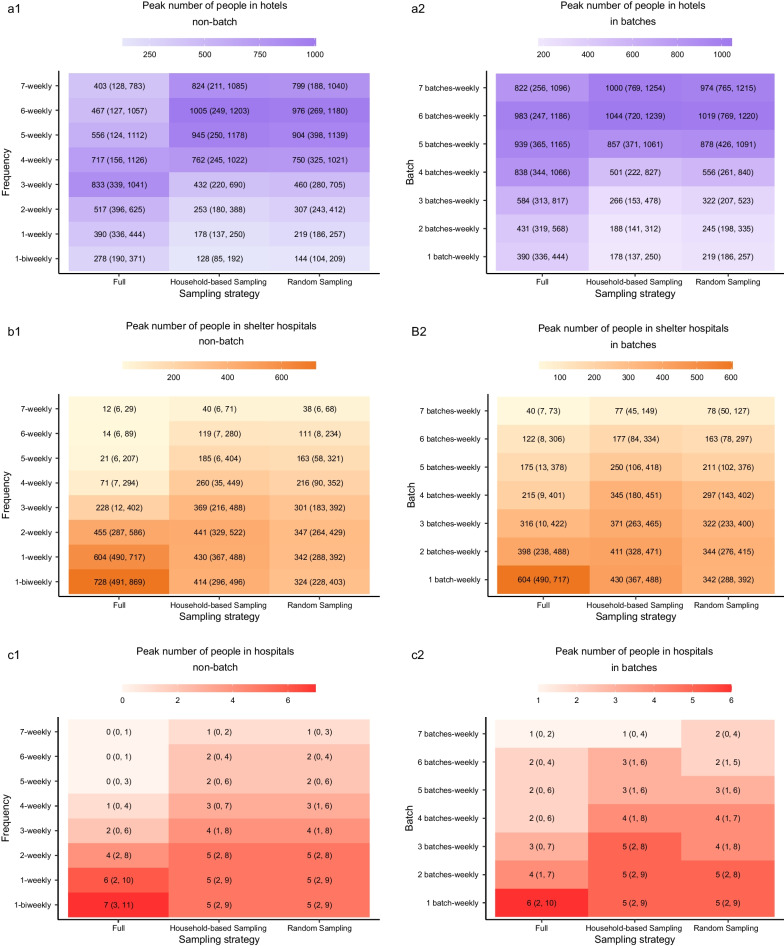
Heatmap showing the peak of quarantine and medical resource consumption of various strategies. **a1** Heatmap of peak number of people in hotels at various frequencies. **a2** Heatmap of peak number of people in hotels in various batches. **b1** Heatmap of peak number of people in shelter hospitals at various frequencies. **b2** Heatmap of peak number of people in shelter hospitals in various batches. **c1** Heatmap of peak number of people in hospitals at various frequencies. **c2** Heatmap of peak number of people in hospitals in various batches. The numbers in brackets were the 95% uncertainty intervals (UIs)

With increasing screening frequency or increasing batches in which a weekly screening was split, the cumulative and peak resource consumption of hotel quarantine increased while the medical resources in shelter hospitals and hospitals decreased (Figs. 5, 6). An exception was the threshold for hotel quarantine. Specifically, when the frequency exceeded three times/week, the cumulative and peak hotel quarantine resource of *Full Screening* decreased, simultaneously the cumulative and peak of quarantine and medical resource for *Full Screening* were less than that for the two sampling strategies. Additionally, the frequency threshold was lower when ${{R}}_{0}$ was lower (Additional file 2: Figs. S10, S11). The resource consumption of hotel quarantine for *Household-based Sample Screening* were generally lower than that for *Random Sample Screening*.

### Results of sensitivity analysis

The results with the different parameters were similar except for ${{R}}_{0}$. The lower the ${{R}}_{0}$, the lower were the $\alpha$ and the more stringent the NPIs, the lower was the cumulative infection rate. The threshold for significantly diminished marginal benefit of frequency was still four times/week for *Full Screening* in our scenarios of different $\alpha$, ${{p}}_{\text{contact}}$, ${{p}}_{\text{casual}}$, and ${{ds}}$, while the threshold is three times/week (${{IR}}_{\text{cum}}$ = 4%) if ${{R}}_{0}$ = 5 and one time/week (${{IR}}_{\text{cum}}$ = 8%) if ${{R}}_{0}$ = 2.5. Besides, the advantage of *Household-based Sampling Screening* and screening in batches remained. In the context of $\alpha$ = 0.5, 0.7, or 0.9: With the frequency of seven times/week, the miss rates of *Household-based Sampling Screening* (26%, 28%, or 30%) were lower than those of *Random Sampling Screening* (31%, 34%, or 38%); ${{IR}}_{\text{cum}}$ s of *Full Screening* seven batches/week (8%, 12%, or 14%) were substantially lower than those of *Full Screening* once per week (83%, 85%, or 87%). The complete results of sensitivity analysis were shown in Additional file 2: Figs. S10–S19.

## Discussion

Large-scale screening is a key measure in China’s control of COVID-19 [29]. A recent study showed that without any interventions, the Omicron variant epidemic in China would result in 5.08 million hospitalizations and 1.55 million deaths [30]. However, mass screening results in numerous social and economic burdens, and unnecessary wastes. This study simulated the epidemic situation and resource consumption of various screening strategies to design nucleic acid screening strategies which balances effectiveness and feasibility.

Our results showed that (1) if the quarantine and medical resources are seriously insufficient, the strict screening strategy—*Full Screening* with high frequency—is necessary. (2) If the testing capacity is quite low, screening the targeted population in many batches is recommended. For example, the tests consumed by *Full Screening* the population once a week in seven batches was considerably less than the tests consumed by *Full Screening* four times/week, although the ${{IR}}_{\text{cum}}$ s of the two strategies were similar. (3) If neither of these resources are sufficient (the most common scenario), the frequency of *Full Screening* does not need to be very high because the marginal effect of increasing frequency on epidemic control decreases with increasing frequency. In the case of ${{R}}_{0}$ = 10, *Full Screening* four times/week is a reasonable compromise. Besides, high-frequency sampling strategies also work under the restriction of testing, quarantine and medical resources. *Household-based Sampling Strategy* is better than *Random Sampling Screening* because the infection rates, miss rates, tests per positive case identified, and hotel resource consumption of the former were all lower. The outbreak mitigation effect of *Household-based Sampling Screening* seven times/week was comparable to *Full Screening* four times/week in our simulation.

We believe that *Household-based Sampling Screening* is potentially promising in practice. Due to the extremely contagious nature of the Omicron variant, family cluster epidemics were common, and the secondary attack rate among households was over 50% [31, 32]. Therefore, the results of screening the individual in each household who has the most contact with the outside can reflect the household’s infection status. *Household-based Sampling Screening* was also convenient to organize in the actual implementation process, and since it screened fewer individuals, it not only reduced the potential risk of disease spread caused by gathering during nucleic acid screening, but also greatly reduced the demand for testing, medical, and other resources during the whole-area screening, ensuring the sampling quality and testing accuracy. In addition, *Household-based Sampling Screening* was conducive to the epidemic control measure implemented—quarantining close contacts—because quarantining infected individuals and their household members together improved the efficiency of transfer and quarantine which in turn helps breaks transmission and improves control the epidemic. *Household-based Sampling Screening* is a form of stratified sampling.

In the future, the prevention and control of COVID-19 will gradually turn to “normalization”, which allows for more flexible “stratification” according to key areas and key populations. A unit consists of a group of people with a similar risk of infection due to close contact with each other. For example, a school, a dormitory room, class, or grade can be considered a unit. A certain number of people from each unit are selected for testing, and if a positive test is found, the entire unit is quarantined.

With a certain frequency of screening, the screening efficacy can be improved by optimizing the timing of screening. We recommend scheduling screenings evenly during a screening cycle. The Shanghai Strategy is an example of testing with a higher frequency at the beginning of a screening cycle, while the Beijing Strategy is closer to evenly distributed screening, the number of infected people in the former being more than that of the latter. Dividing once only screening into multiple batches is an extended way of arranging the tests evenly during a certain time period. The more batches, the more even the distribution. Furthermore, screening above the testing capacity is likely to extend the turnaround time of transferring people to the quarantine or medical places, reduce the quality of sampling and testing, and influence the efficiency of close contact tracing and quarantine [20]. So, screening in batches—which can effectively control the epidemic with a small amount of testing—is worth pursuing.

This study has several limitations. First, reinfection with COVID-19 was not considered. Previous studies have indicated that the reinfection rate of COVID-19 is approximately 0.22–0.32% [33], which is relatively low. We only simulated the transmission of COVID-19 during a period of 100 days and, therefore, ignored the occurrence of reinfection of COVID-19 cases in our study. Second, the effects of the vaccine were not considered. Due to the immune evasion of the Omicron variant, the present vaccine significantly prevents severe cases and deaths, but is not enough to prevent morbidity [25, 34]. Thus, ignoring the effect of the vaccine did not affect the main results of this study. Third, we did not directly account for the effect of variation in viral load on the testing results; however, the effect of variation in viral load was considered indirectly by accounting for variations in the probability of testing at different times after infection. We only considered the heterogeneity in the rate of severe illness among different age groups and did not address differences in susceptibility. Due to the highly contagious nature of the Omicron variant and the fact that family members were fully exposed in their households during the simulation period under “lockdown measures”, all family members were more likely to be infected if one family member been infected. This assumption did not significantly alter the conclusions of the present study.

## Conclusions

Intermediate screening (such as four times/week for a fast-spreading outbreak), screening in batches, and *Household-based Sampling Screening* are recommended with the goal of mitigating the outbreak and restricting the consumption of resources. The specific strategy design should be determined according to the virus transmission feature and actual resource constraints considering cost-effectiveness, feasibility and sustainability.

## Supplementary Information

**Additional file 1: Supplements of the methods.** **Text S1.** Stochastic network-based dynamic model (P2). **Text S2.** Community network structure (P7). **Table S1.** Parameters related to disease transmission and control (P4). **Table S2.** Screening strategy (P6). **Table S3.** False negative rate of nucleic acid testing (1-test sensitivity) (P8). **Table S4.** Network parameters (P10). **Table S5.** Sensitivity analysis parameters (P11). **Fig. S1.** Compartmental structure used to describe the progression of disease states (P11). **Fig. S2.** A. Random network degree distribution; B. Network degree distribution after increasing social distance (P12).

**Additional file 2: Supplements of the results.** **Fig. S3.** The number of infections in 100 days for each strategy (P2). **Fig. S4.** The cumulative infection rates of 1000 simulations (P3). **Fig. S5.** The number of people quarantined in the hotel in 100 days for each strategy (P4). **Fig. S6.** The number of people quarantined in the shelter in 100 days for each strategy (P5). **Fig. S7.** The number of hospitalized cases in 100 days for each strategy (P6). **Fig. S8.** Cumulative infection rate, miss rate, and tests per positive of screening at different times (P7). **Fig. S9.** Cumulative and the maximum number of quarantine and hospitalized people of screening at different times (P7). **Fig. S10.** Cumulative infection rate, miss rate, tests per positive, the number of quarantining and hospitalization when ${{R}}_{0}$ = 2.5 (P8). **Fig. S11.** Cumulative infection rate, miss rate, tests per positive, the number of quarantining and hospitalization when ${{R}}_{0}$ = 5 (P9). **Fig. S12.** Cumulative infection rate, miss rate, tests per positive, the number of quarantining and hospitalization when $\alpha$ = 0.5 (P10). **Fig. S13.** Cumulative infection rate, miss rate, tests per positive, the number of quarantining and hospitalization when $\alpha$ = 0.7 (P11). **Fig. S14.** Cumulative infection rate, miss rate, tests per positive, the number of quarantining and hospitalization when ${{ds}}$ = 0.7213475 (P12). **Fig. S15.** Cumulative infection rate, miss rate, tests per positive, the number of quarantining and hospitalization when ${{ds}}$ = 3.4760595 (P13). **Fig. S16.** Cumulative infection rate, miss rate, tests per positive, the number of quarantining and hospitalization when ${{p}}_{{\text{ca}}{\text{sual}}}$ = 0.1 (P14). **Fig. S17.** Cumulative infection rate, miss rate, tests per positive, the number of quarantining and hospitalization when ${{p}}_{\text{casual}}$ = 0.2 (P15). **Fig. S18.** Cumulative infection rate, miss rate, tests per positive, the number of quarantining and hospitalization when ${{p}}_{\text{contact}}$ = 0.85 (P16). **Fig. S19.** Cumulative infection rate, miss rate, tests per positive, the number of quarantining and hospitalization when ${{p}}_{\text{contact}}$ = 0.95 (P17).

## Data Availability

The datasets generated and analyzed during the current study are available in the GitHub repository (https://github.com/strongermw/screening-strategies).

## Abbreviations

COVID-19: Coronavirus disease 2019
SARS-CoV-2: Severe acute respiratory syndrome coronavirus 2
95% UI: 95% Uncertainty interval
$\alpha$: Proportion of asymptomatic infected
NPIs: Non-pharmacological interventions
${{p}}_{\text{casual}}$: Global transmission parameter
$${{ds}}$$: Distancing scale
${{R}}_{\text{t}}$: Effective reproduction number
${{IR}}_{\text{cum}}$: Cumulative infection rate
${{R}}_{0}$: Basic reproduction number
${{p}}_{\text{contact}}$: Probability of close contacts being successfully traced
