# Supplementary material for: Optimizing the nucleic acid screening strategy to mitigate regional outbreaks of SARS-CoV-2 Omicron variant in China: a modeling study
Source: Infect Dis Poverty. 2023 Jan 16;12:1. doi: 10.1186/s40249-022-01049-w (PMC9841147; doi:10.1186/s40249-022-01049-w)
Supplement: Supplementary file 1 — Additional file 1: Supplements of the methods. Text S1. Stochastic network-based dynamic model (P2). Text S2. Community network structure (P7). Table S1. Parameters related to disease transmission and control (P4). Table S2. Screening strategy (P6). Table S3. False negative rate of nucleic acid testing (1-test sensitivity) (P8). Table S4. Network parameters (P10). Table S5. Sensitivity analysis parameters (P11). Fig. S1. Compartmental structure used to describe the progression of disease states (P11). Fig. S2. A. Random network degree distribution; B. Network degree distribution after increasing social distance (P12). [file 40249_2022_1049_MOESM1_ESM.docx]

**Additional file 1**

**Contexts**

**Text**

Text S1 Stochastic network-based dynamic model (P2)

Text S2 Community network structure (P7)

**Table**

Table S1 Parameters related to disease transmission and control (P4)

Table S2 Screening strategy (P6)

Table S3 False negative rate of nucleic acid testing (1-test sensitivity) (P8)

Table S4 Network parameters (P10)

Table S5 Sensitivity analysis parameters (P11)

**Figure**

Fig. S1 Compartmental structure used to describe the progression of disease states (P11)

Fig. S2 A. Random network degree distribution; B. Network degree distribution after increasing social distance (P12)

**Reference (13)**

# Text S1 Stochastic network-based dynamic model

## Text S1.1 Compartments

To simulate the transmission of SARS-CoV-2 in real word, we established an extended $\text{SE}\text{I}_{\text{pre}}\text{I}_{\text{sym}}\text{I}_{\text{asym}}\text{HRF}\text{ }$model based on the classic SEIR model (Fig 1), including susceptible ($\text{S}$), exposed ($\text{E}$), pre-symptomatic ($\text{I}_{\text{pre}}$), symptomatic ($\text{I}_{\text{sym}}$), asymptomatic ($\text{I}_{\text{asym}}$), hospitalized ($\text{H}$), recovered ($\text{R}$) and fatal ($\text{F}$). Susceptible individuals ($\text{S}$) become exposed ($\text{E}$, non-infectious) after being infected once contacted with infectious individuals ($\text{I}_{\text{pre}}\text{, }\text{I}_{\text{asym}}\text{ or }\text{I}_{\text{sym}}$). After 1/σ days of latent period, they enter the pre-symptomatic stage ($\text{I}_{\text{pre}}$), in which the infected can infect others but asymptomatic, and this period was called as the pre-symptomatic stage. Then some of them (proportion 1-α) developed symptoms (symptomatic infected, $\text{I}_{\text{sym}}$), among which some were ill so severe that they had to be hospitalized ($\text{H}$), and a small fraction ($\text{f}$) of the hospitalized died ($\text{F}$). The probability of developing severe illness and dying from illness of infected were different from age groups (See $\text{h}_{\text{i}}$ in Table S1 for details). The remaining $\text{I}_{\text{pre}}$ (proportion α) were always asymptomatic $\text{(}\text{I}_{\text{asym}}$), and recover ($\text{R}$) after$\text{ 1/}\text{γ}_{\text{A}}$ days, just as the mildly infected (proportion 1-*h*), after 1/γ days in the symptomatic period. Here, we assumed that the infected would develop immunity to SARS-CoV-2 after recovery, and would not be reinfected during the simulation period.

During the simulation, intervention measures (testing, tracing, and isolation) would be implemented once the total infection rate reached a threshold (5/10,000 in the main text used). The effect of isolation was modeled by introducing compartments that represent quarantined individuals, and an individual can be isolated in any disease states (we set a corresponding isolation compartment for each disease state except for *H* and *F*). The individuals tested positive, and their family members and close contacts outside their household would be isolated and entered the corresponding isolation compartments. Isolated individuals followed the same disease progression through the disease states, so that isolated symptomatic individuals would be hospitalized if they were ill severely during the isolation period. We assumed that isolated individuals cannot contact others, therefore, susceptible isolated ($\text{Q}_{\text{S}}$) would not be infected, either infectious individuals isolated ($\text{Q}_{\text{pre}}\text{, }\text{Q}_{\text{sym}}\text{ or }\text{Q}_{\text{asym}}$) infect others. After in quarantine for the isolation period (14 days in this study), the isolated individuals transition to the nonquarantine compartments corresponding to their disease states at that time.

The parameter descriptions and values can be seen in Table S1.

## Text S1.2 Dynamic transmission

We implemented our simulation with the SEIRS+ package with modification [1]. The dynamic transmission was simulated using the Gillespie algorithm. Simply, we computed the ‘propensity’ of the possible events (i.e., the expected amount of time until a given event will take place) for all nodes at each time step, as an alternative to solving different equation systems for deterministic models. These propensities were then used to compute the probabilities of all possible state events normalized across the entire population. In each time step, a random node and corresponding transition were selected to execute according to these probabilities.

There were two contact modes that both can lead to exposure of susceptible individuals and disease transmission events: close contacts (connections defined by the network structure, called local transmission) and casual contacts (called global transmission). Local transmission refers to transmission events that occur due to repeated, continuous or close contact, such as contacts between family members; global transmission refers to the occurrence of incidents due to accidental contact between individuals (such as contacts happened in public places) without clear network connections between individuals. The proportion of local and global transmission was defined using the locality parameter $\text{p}_{\text{casual}}$: when $\text{p}_{\text{casual}}\text{=0}$, individuals interact only with close contacts, while $\text{p}_{\text{casual}}\text{=1}$ represents a uniformly mixed population.

Each individual $\text{i}$ has a state $\text{X}^{\text{(i)}}$, The propensities of state transitions for a given node can be described by the following equations:

$$\text{P}_{\text{i}}\left( \text{S→E} \right)\text{=}\text{a}^{\text{(i)}}\left[ \text{p}_{\text{casual}}\left( \frac{\bar{\text{β}}\left( \text{I}_{\text{pre}}\text{+}\text{I}_{\text{asym}}\text{+}\text{I}_{\text{sym}} \right)}{\text{N}} \right)\text{+(1-}\text{p}_{\text{casual}}\text{)(}\frac{\sum_{\text{j}\text{∈}\text{C}_{\text{G}}^{\left( \text{i} \right)}} \text{δ}^{\text{ji}}\text{(}\text{β}_{\text{j}}\text{1}_{\text{X}^{\text{(j)}}\text{∈}\text{\{}\text{I}_{\text{pre}}\text{, }\text{I}_{\text{asym}}\text{,}\text{I}_{\text{sym}}\text{\}}}\text{)}}{\left| \text{C}_{\text{G}}^{\left( \text{i} \right)} \right|}\text{)} \right]$$

$$\text{P}_{\text{i}}\left( \text{E→}\text{I}_{\text{pre}} \right)\text{=}\text{σ}_{\text{i}}\text{1}_{\text{X}^{\text{(i)}}}\text{=E}$$

$$\text{P}_{\text{i}}\left( \text{I}_{\text{pre}}\text{→}\text{I}_{\text{sym}} \right)\text{=}\left( \text{1-α} \right)\text{λ}_{\text{i}}\text{1}_{\text{X}^{\text{(i)}}}\text{=}\text{I}_{\text{pre}}$$

$$\text{P}_{\text{i}}\left( \text{I}_{\text{pre}}\text{→}\text{I}_{\text{asym}} \right)\text{=α}\text{λ}_{\text{i}}\text{1}_{\text{X}^{\text{(i)}}}\text{=}\text{I}_{\text{pre}}$$

$$\text{P}_{\text{i}}\left( \text{I}_{\text{sym}}\text{→R} \right)\text{=(1-}\text{h}_{\text{i}}\text{)}\text{γ}_{\text{i}}\text{1}_{\text{X}^{\text{(i)}}}\text{=}\text{I}_{\text{sym}}$$

$$\text{P}_{\text{i}}\left( \text{I}_{\text{sym}}\text{→H} \right)\text{=}\text{h}_{\text{i}}\text{η}_{\text{i}}\text{1}_{\text{X}^{\text{(i)}}}\text{=}\text{I}_{\text{sym}}$$

$$\text{P}_{\text{i}}\left( \text{I}_{\text{asym}}\text{→R} \right)\text{=}\text{γ}_{\text{A}_{\text{i}}}\text{1}_{\text{X}^{\text{(i)}}}\text{=}\text{I}_{\text{asym}}$$

$$\text{P}_{\text{i}}\left( \text{H→R} \right)\text{=}{\left( \text{1-}\text{f}_{\text{i}} \right)\text{γ}}_{\text{H}_{\text{i}}}\text{1}_{\text{X}^{\text{(i)}}}\text{=H}$$

$$\text{P}_{\text{i}}\left( \text{H→F} \right)\text{=}\text{f}_{\text{i}}\text{μ}_{\text{H}_{\text{i}}}\text{1}_{\text{X}^{\text{(i)}}}\text{=H}$$

Under isolation, susceptible individuals cannot become infected ($\text{Q}_{\text{S}}$ cannot transfer to $\text{Q}_{\text{E}}$), the transfer equations between other isolation compartments were similar as above. The meanings of some parameters in the equations (the others can be seen in Table S1):

- $\text{a}^{\text{(i)}}$: the susceptibility of the susceptible individual $\text{i}$;
- $\text{p}_{\text{casual}}$: a network locality parameter, determining the relative frequency and weight of transmission among local and global contacts in the model;
- $\bar{\text{β}}$: the mean transmissibility of the symptomatic individuals;
- $\text{β}_{\text{j}}$: the transmissibility of the symptomatic individual *j*;
- $\text{C}_{\text{G}}^{\left( \text{i} \right)}$: the set of close contacts for individual $\text{i}$;
- $\text{1}_{\text{X}^{\text{(j)}}}$: an indicator function that takes the value 1 when the state *X^(j)^* of the node j is one of the infectious states and 0 otherwise.

**Table S1 Parameters related to disease transmission and control**

| **Parameters and description** | **Value/range** | **Reference** |
| --- | --- | --- |
| $\text{α}$: proportion of asymptomatic infection | 0$\text{.}$9 | [2] |
| $\text{R}_{\text{0}}$: the expected number of secondary cases that an individual generates when infectious in a  fully susceptible population | Gamma (10, 0$\text{.}$35) | [3](Mean), Assumed (CV) |
| $\text{σ}$: time in exposed state | gamma (3$\text{.}$2,0$\text{.}$6875) | [4] |
| $\text{λ}$: time in pre-symptomatic infectious state | gamma (3$\text{.}$83, 0$\text{.}$5979) | [5] |
| $\text{γ, }\text{γ}_{\text{A}}$: (a)symptomatic periods | gamma (6$\text{.}$87, 0$\text{.}$7584) | [6] |
| Infectious Period | $\text{λ}$+$\text{γ}$ | / |
| $\text{β}$: transmission rate of symptomatic infected | 1/infectious Period * R0 | / |
| Pre-symptomatic transmissibility (relative to symptomatic transmissibility) | 0$\text{.}$5 | [7] |
| Asymptomatic transmissibility (relative to symptomatic transmissibility) | 0$\text{.}$5 | [7] |
| $\text{1/η}$: time in symptomatic state before entering hospitalized state for those with severe cases | gamma (4$\text{.}$0, 0$\text{.}$9027) | [8] |
| $\text{1/}\text{γ}_{\text{H}}$: time in hospitalized state for those with non-fatal cases | gamma (16$\text{.}$0, 0$\text{.}$4178) | [8] |
| $\text{1/}\text{μ}_{\text{H}}$: time in hospitalized state for those with fatal cases | gamma (12$\text{.}$0, 0$\text{.}$9545) | [8] |
| $\text{p}_{\text{symtest}}$: proportion of active testing after symptomatic infection developed symptoms | 0$\text{.}$5 | Assumed |
| Compliance with isolation and testing | 100% | Assumed |
| $\text{p}_{\text{contact}}$: proportion of close contacts successfully traced | 0$\text{.}$9 | Assumed |
| $\text{h}_{\text{i}}$: hospitalization rates by age group | 0-9: 0$\text{.}$0000  10-19: 0$\text{.}$0004,  20-29: 0$\text{.}$0104,  30-39: 0$\text{.}$0343,  40-49: 0$\text{.}$0425,  50-59: 0$\text{.}$0816,  60-69: 0$\text{.}$118,  70-79: 0$\text{.}$166,  80+: 0$\text{.}$184 | [9] |
| $\text{f}_{\text{i}}$: mortality due to illness in hospitalized patients in different age groups | 0-9: 0$\text{.}$0000,  10-19: 0$\text{.}$3627,  20-29: 0$\text{.}$0577,  30-39: 0$\text{.}$0426,  40-49: 0$\text{.}$0694,  50-59: 0$\text{.}$1532,  60-69: 0$\text{.}$3381,  70-79: 0$\text{.}$5187,  80+: 0$\text{.}$7283 | [9] |

## Text S1.3 Testing, tracing and isolation

### Text S1.3.1 Testing

Considering different sampling methods, screening frequency and sampling time, a total of 87 scenarios were designed, and we set the full screening as the baseline scenario to compare the epidemic development, screening efficiency and medical resource pressure under different scenarios (See Table S2 for detailed scenarios). All scenarios take 14 days or seven days as a testing cycle, and specify the specific testing date. We assumed that a certain proportion ($\text{p}_{\text{symtest}}$) of symptomatic infected individuals would seek testing institutions for testing proactively after developed symptoms, and the testing could be performed regardless whether it was a testing day. However, other tests, including for the close contacts, were only carried out on the testing days.

On the testing days, those meeting one of the following conditions were excluded from being tested (the individuals to be tested determined by the sampling scenario in Table S2): (1) positive test results in the past; (2) hospitalized (confirmed due to COVID-19); (3) recovered from COVID-19 disease state; (4) dead.

The testing sensitivity (1 - false negative rate) varies depending on the disease states (exposed, pre-symptomatic, symptomatic or asymptomatic) and the amount of time the individual had spent in a given state. See Table S3 for the testing sensitivity of exposed, pre-symptomatic, symptomatic, and asymptomatic infected individuals at different times in the corresponding states[10, 11].

**Table S2** **Designs of screening strategies**

| **1. Sampling scenarios—determining screening populations** | | | |
| --- | --- | --- | --- |
| **Scenario** | | **Description of whole-area screening object** | |
| $\text{S}_{\text{i}}$（$\text{i=1}\text{,}\text{ 2}\text{,}\text{ 3}$） | | 1: Full screening (N= total community population);  2: Stratified sampling by households（the individual in each family with the most connections to the outside is selected for testing, and repeats the testing of these individuals，N= number of households），  3: Random sampling（random sampling of the population according to the household sampling proportion (total community population/number of households)，and repeats the testing of these individuals，N= number of households） | |
| **2. Screening strategies - implement strategies for three types of screening populations respectively** | | | |
|  | **Scenario** | **Description of screening strategies** | **Testing date within 14 days** |
| **Census** | $\text{S}_{\text{i}}$-C1 | 7 times per week | (1, 2, 3, 4, 5, 6, 7, 8, 9, 10, 11, 12, 13, 14) |
|  | $\text{S}_{\text{i}}$-C2 | 6 times per week | (1, 2, 3, 4, 5, 6, 8, 9, 10, 11, 12, 13) |
|  | $\text{S}_{\text{i}}$-C3 | 5 times per week | (1, 2, 3, 4, 5, 8, 9, 10, 11, 12) |
|  | $\text{S}_{\text{i}}$-C4 | testing on first seven days, days 10 and 14 | (1, 2, 3, 4, 5, 6, 7, 10, 14) - Shenzhen, China |
|  | $\text{S}_{\text{i}}$-C5 | 4 times per week | (1, 2, 3, 4, 8, 9, 10, 11) |
|  | $\text{S}_{\text{i}}$-C6 | 3 times per week | (1, 2, 3, 8, 9, 10) |
|  | $\text{S}_{\text{i}}$-C7 | testing on days 1, 2, 4, 7 and 14 | (1, 2, 4, 7, 14) -Shanghai, China |
|  | $\text{S}_{\text{i}}$-C8 | testing on first seven days, days 10 and 14 | (1, 4, 7, 10, 14) - Beijing, China |
|  | $\text{S}_{\text{i}}$-C9 | twice per week | (1, 2, 8, 9) |
|  | $\text{S}_{\text{i}}$-C10 | once per week | (1, 8, ...) |
|  | $\text{S}_{\text{i}}$-C11 | once every two weeks | (1, 15, ...) |
| **Census’ time point distributions** | $\text{S}_{\text{i}}$-CT1 | 4 times per week | Even distribution (1, 3, 5, 7, 8, 10, 12, 14) |
|  | $\text{S}_{\text{i}}$-CT2 |  | Front distribution (1, 2, 3, 4, 8, 9, 10, 11) |
|  | $\text{S}_{\text{i}}$-CT3 |  | Center distribution (3, 4, 5, 6, 10, 11, 12, 13) |
|  | $\text{S}_{\text{i}}$-CT 4 |  | Rearward distribution (4, 5, 6, 7, 11, 12, 13, 14) |
|  | $\text{S}_{\text{i}}$-CT 5 | 3 times per week | Even distribution (1, 4, 7, 8, 11, 14) |
|  | $\text{S}_{\text{i}}$-CT 6 |  | Front distribution (1, 2, 3, 8, 9, 10) |
|  | $\text{S}_{\text{i}}$-CT 7 |  | Center distribution (3, 4, 5, 10, 11, 12) |
|  | $\text{S}_{\text{i}}$-CT 8 |  | Rearward distribution (5, 6, 7, 12, 13, 14) |
| **Testing in batches** | $\text{S}_{\text{i}}$-B1 | 6 times per week (N/7 people are tested each time, and complete full screening in one week) | (1, 2, 3, 4, 5, 6, 7, 8, 9, 10, 11, 12, 13, 14) |
|  | $\text{S}_{\text{i}}$-B2 | 5 times per week (N/5 people are tested each time, and complete full screening in one week) | (1, 2, 3, 4, 5, 6, 8, 9, 10, 11, 12, 13) |
|  | $\text{S}_{\text{i}}$-B3 | 4 times per week (N/4 people are tested each time, and complete full screening in one week) | (1, 2, 3, 4, 5, 8, 9, 10, 11, 12) |
|  | $\text{S}_{\text{i}}$-B4 | 3 times per week (N/3 people are tested each time, and complete full screening in one week) | (1, 2, 3, 4, 8, 9, 10, 11) |
|  | $\text{S}_{\text{i}}$-B5 | twice per week (N/2 people are tested each time, and complete full screening in one week) | (1, 2, 3, 8, 9, 10) |
|  | $\text{S}_{\text{i}}$-B6 | once per week (N people are tested each time, and complete full screening in one week) | (1, 2, 8, 9) |
| **Testing in batches’ time point distribution** | $\text{S}_{\text{i}}$-BT1 | 4 times per week  (N/4 people are tested each time, and complete full screening in one week) | Even distribution (1, 3, 5, 7, 8, 10, 12, 14) |
|  | $\text{S}_{\text{i}}$-BT2 |  | Front distribution (1, 2, 3, 4, 8, 9, 10, 11) |
|  | $\text{S}_{\text{i}}$-BT3 |  | Center distribution (3, 4, 5, 6, 10, 11, 12, 13) |
|  | $\text{S}_{\text{i}}$-BT4 |  | Rearward distribution (4, 5, 6, 7, 11, 12, 13, 14) |
|  | $\text{S}_{\text{i}}$-BT5 | 3 times per week  (N/3 people are tested each time, and complete full screening in one week) | Even distribution (1, 4, 7, 8, 11, 14) |
|  | $\text{S}_{\text{i}}$-BT6 |  | Front distribution (1, 2, 3, 8, 9, 10) |
|  | $\text{S}_{\text{i}}$-BT7 |  | Center distribution (3, 4, 5, 10, 11, 12) |
|  | $\text{S}_{\text{i}}$-BT8 |  | Rearward distribution (5, 6, 7, 12, 13, 14) |

**Table S3 False** **negative rate of nucleic acid testing of different infection states**

| **Infection states** | **temporal false negative rates** |
| --- | --- |
| $\text{E}$&$\text{Q}_{\text{E}}$ | 0: 1$\text{.}$00, 1: 1$\text{.}$00, 2: 1$\text{.}$00, 3: 1$\text{.}$00 |
| $\text{I}_{\text{pre}}$&$\text{Q}_{\text{pre}}$ | 0: 0$\text{.}$25, 1: 0$\text{.}$25, 2: 0$\text{.}$22 |
| $\text{I}_{\text{asym}}$&$\text{Q}_{\text{asym}}$  $\text{I}_{\text{sym}}$&$\text{Q}_{\text{sym}}$ | 0: 0$\text{.}$19, 1: 0$\text{.}$16, 2: 0$\text{.}$16, 3: 0$\text{.}$17, 4: 0$\text{.}$19,  5: 0$\text{.}$22, 6: 0$\text{.}$26, 7: 0$\text{.}$29, 8: 0$\text{.}$34, 9: 0$\text{.}$38,  10: 0$\text{.}$43, 11: 0$\text{.}$48, 12: 0$\text{.}$52, 13: 0$\text{.}$57, 14: 0$\text{.}$62,  15: 0$\text{.}$66, 16: 0$\text{.}$70, 17: 0$\text{.}$76, 18: 0$\text{.}$79, 19: 0$\text{.}$82,  20: 0$\text{.}$85, 21: 0$\text{.}$88, 22: 0$\text{.}$90, 23: 0$\text{.}$92, 24: 0$\text{.}$93,  25: 0$\text{.}$95, 26: 0$\text{.}$96, 27: 0$\text{.}$97, 28: 0$\text{.}$97, 29: 0$\text{.}$98,  30: 0$\text{.}$98, 31: 0$\text{.}$99 |

### Text S1.3.2 Tracing and isolation

We assumed 100% compliance with testing and isolation policies for all individuals (in China, it is included in the *Infectious Disease Prevention and Control Law* for management); there was a 1-day delay between individuals been tested and receiving the test reports. Individuals who test positive would be immediately isolated and their close contacts would be traced, we assumed that a proportion of $\text{p}_{\text{contact}}$ (90% in our simulation) of contacts were traced successfully. Successfully traced close contacts would be immediately isolated, but the tracing process took 1 day, that was, there was a 1-day delay from reporting an infection case to isolating his/her close contacts.

The isolated individuals would not contact others (A1.1). As recommended by the CCDC, the isolated individuals would be quarantined for 14 days. In the model, we assumed that the traced close contacts (with negative test results) entered hotels for isolation, asymptomatic and mild patients (with positive test results) entered shelter hospitals, while severe patients were admitted to hospitals for treatments.

# Text S2 Community network structure

We first assumed a community containing 2000 residents (nodes on the network), contacts among residents were represented by connections on the network. Then, we simulated the transmission of SARS-CoV-2 for each screening strategy. Accounting for the effects of randomness in network structure and transmission dynamics, we repeated the above process 1000 times.

## Text S2.1 Network structure

Based on the age structure distribution of China's urban population (Table S4), each member in the community was assigned to a certain age group (0–9, 10–19, ... 70–79, 80+), and then they were assigned to different families according to the size distribution of families (Table S4)[12].

We assumed that all members in one family were fully connected, and individuals among different families were randomly connected, which depended on the age groups. In this study, we randomly connected the nodes based on the contact rates in Shanghai surveyed before the COVID-19 epidemic (Table S4)[13].

For our simulation, once the total infection rate of the community exceeds a threshold (0$\text{.}$0005 in our research), intervention measures would be implemented, including increasing social distancing, nucleic acid testing, and tracing close contacts. Increasing social distancing would cause some connections been cut off. We assumed that only the connections between different families were cut off, while family members remain fully connected. We implemented this step using the parameter $\text{ds}$ in combination with an exponentially distributed random number, and approximately $\text{100(1-exp(-1/}\text{ds}\text{))\%}$ of individuals would be cut off. Therefore, more smaller the value of $\text{ds}$ was, more connections outside households would be cut off. Fig 1.B showed the network with $\text{ds}$=1$\text{.}$442695, which corresponds to about 50% of individuals would have no connection with members outside his/her family. The degree distributions before and after interventions were shown in Fig S2.

**Table S4 Network parameters**

| **Parameters** | **Description** | **Value** | **Source** |
| --- | --- | --- | --- |
| household size distribution | The proportion of different family sizes | 1: 0$\text{.}$2837034254,  2: 0$\text{.}$3455088159,  3: 0$\text{.}$2238563271,  4: 0$\text{.}$0845357644,  5: 0$\text{.}$0466034563,  6: 0$\text{.}$0126443446,  7: 0$\text{.}$0021816290,  8: 0$\text{.}$0006086290,  9: 0$\text{.}$0001926461,  10: 0$\text{.}$0001649623 | [12] |
| age distribution | The proportion of the population in different age groups | 0–9: 0$\text{.}$1093,  10–19: 0$\text{.}$1043,  20–29: 0$\text{.}$1437,  30–39: 0$\text{.}$1881,  40–49: 0$\text{.}$1552,  50–59: 0$\text{.}$1439,  60-69: 0$\text{.}$0908,  70–79: 0$\text{.}$0437,  80+: 0$\text{.}$0210 | [12] |
| layer definitions | Contact rates for different age groups | 0–9: 11$\text{.}$6 (9$\text{.}$2, 14$\text{.}$3),  10–19:27 (23$\text{.}$1,30$\text{.}$7),  20–39:22$\text{.}$4 (19$\text{.}$8,25$\text{.}$9),  40–59:19$\text{.}$9 (17$\text{.}$7,23$\text{.}$3),  60+:12$\text{.}$6 (10$\text{.}$8, 14$\text{.}$7) | [13] |
| $\text{p}_{\text{casual}}$ | The probability of interactions being with incidental or casual contacts outside their set of close contacts | 0$\text{.}$05 | Assumed |
| distancing scale | Generate a quarantine version of the network where a majority of the out-of-household edges have been removed | 1$\text{.}$442695 (about 50% of individuals would have no connection with members outside his/her family) | Assumed |

**Table S5 Sensitivity analysis parameters**

| **Parameters** | **Default value** | **Sensitivity analysis** |
| --- | --- | --- |
| $\text{R}_{\text{0}}$: basic reproduction number | 10 | 5, 2$\text{.}$5 |
| $\text{α}$: proportion of asymptomatic infected | 0$\text{.}$9 | 0$\text{.}$5, 0$\text{.}$7 |
| $\text{p}_{\text{contact}}$: proportion of close contacts successfully traced | 0$\text{.}$9 | 0$\text{.}$85, 0$\text{.}$95 |
| $\text{p}_{\text{casual}}$: The probability of interactions being with incidental or casual contacts outside their set of close contacts | 0$\text{.}$05 | 0$\text{.}$1, 0$\text{.}$2 |
| distancing scale: generate a quarantine version of the network where a majority of the out-of-household edges have been removed | 1$\text{.}$442695 (about 50% of individuals would have no connection with members outside his/her family) | 0$\text{.}$7213415 (about 75% of individuals would have no connection with members outside his/her family)，3$\text{.}$4760595 (about 25% of individuals would have no connection with members outside his/her family) |

**
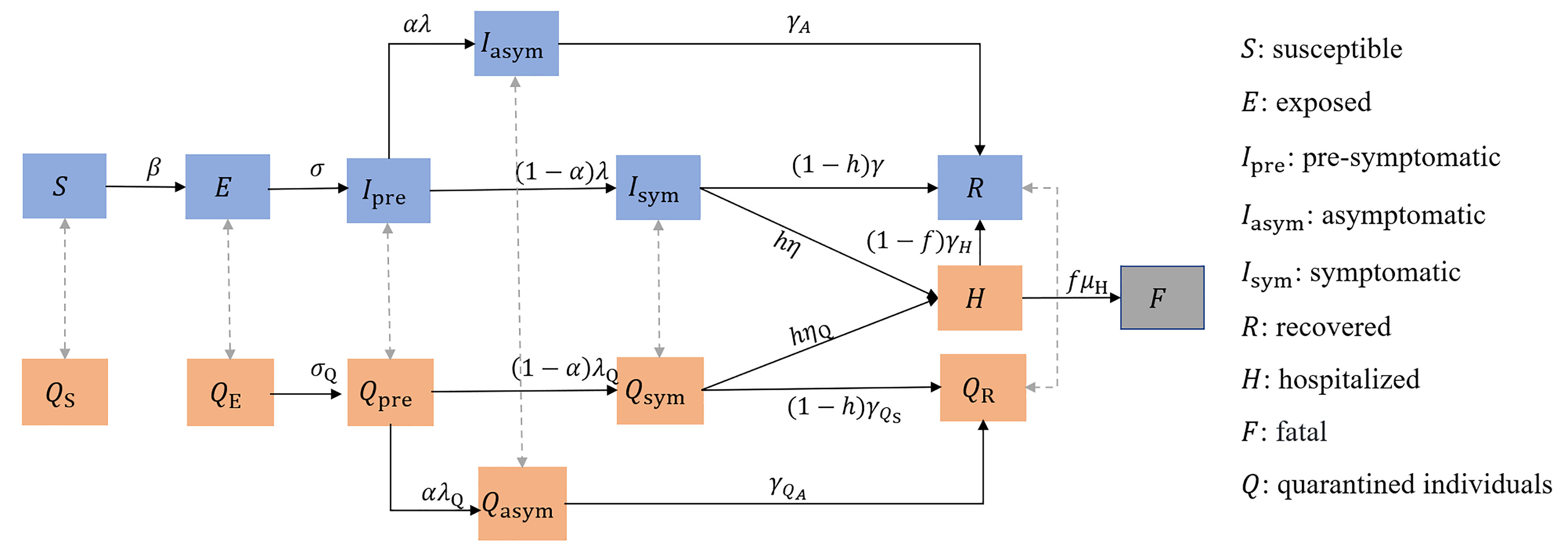
**

**Fig. S1 Compartmental structure used to describe the progression of disease states**


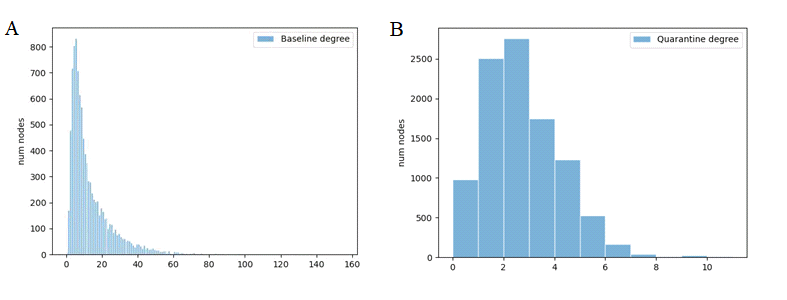


**Fig. S2 A. Random network degree distribution; B. Network degree distribution after increasing social distance**

# References

1. National Bureau of Statistics of China. China Statistical Yearbook 2021. 2021. http://www.stats.gov.cn/tjsj/ndsj/2021/indexch.htm. Accessed 1 Jun 2022.

2. NetEase News. The epidemic in Shanghai has mutated, and the proportion of asymptomatic infections is as high as 92%. 2022. https://www.163.com/dy/article/H26L0FGT0534CJTD.html. Accessed 7 June 2022.

3. Davido B, Dumas L, Rottman M. Modelling the Omicron wave in France in early 2022: Balancing herd immunity with protecting the most vulnerable. J Travel Med. 2022;29(3):taac005.

4. Backer JA, Eggink D, Andeweg SP, Veldhuijzen IK, van Maarseveen N, Vermaas K, et al. Shorter serial intervals in SARS-CoV-2 cases with Omicron BA.1 variant compared with Delta variant, the Netherlands, 13 to 26 December 2021. Euro Surveill. 2022;27(6):2200042.

5. Ma X, Wu K, Li Y, Li S, Cao L, Xie H, et al. Contact tracing period and epidemiological characteristics of an outbreak of the SARS-CoV-2 Delta variant in Guangzhou. Int J Infect Dis. 2022;117:18-23.

6. Menni C, Valdes AM, Polidori L, Antonelli M, Penamakuri S, Nogal A, et al. Symptom prevalence, duration, and risk of hospital admission in individuals infected with SARS-CoV-2 during periods of omicron and delta variant dominance: a prospective observational study from the ZOE COVID Study. Lancet. 2022;399(10335):1618-24.

7. McEvoy D, McAloon C, Collins A, Hunt K, Butler F, Byrne A, et al. Relative infectiousness of asymptomatic SARS-CoV-2 infected persons compared with symptomatic individuals: a rapid scoping review. BMJ Open. 2021;11(5):e042354.

8. Shen Y, Zheng F, Sun D, Ling Y, Chen J, Li F, et al. Epidemiology and clinical course of COVID-19 in Shanghai, China. Emerg Microbes Infect. 2020;9(1):1537-45.

9. Verity R, Okell LC, Dorigatti I, Winskill P, Whittaker C, Imai N, et al. Estimates of the severity of coronavirus disease 2019: a model-based analysis. Lancet Infect Dis. 2020;20(6):669-77.

10. Kucirka LM, Lauer SA, Laeyendecker O, Boon D, Lessler J. Variation in False-Negative Rate of Reverse Transcriptase Polymerase Chain Reaction-Based SARS-CoV-2 Tests by Time Since Exposure. Ann Intern Med. 2020;173(4):262-7.

11. Wikramaratna P, Paton RS, Ghafari M, Lourenço J. Estimating false-negative detection rate of SARS-CoV-2 by RT-PCR. medRxiv. 2020:doi: 10.2807/1560-7917.ES.2020.25.50.2000568.

12. China Statistical Yearbook 2021. 2021. http://www.stats.gov.cn/tjsj/ndsj/2021/indexch.htm. Accessed 1 Sept.

13. Zhang J, Litvinova M, Liang Y, Wang Y, Wang W, Zhao S, et al. Changes in contact patterns shape the dynamics of the COVID-19 outbreak in China. Science. 2020;368(6498):1481-6.
