# Supplementary material for: Optimizing the nucleic acid screening strategy to mitigate regional outbreaks of SARS-CoV-2 Omicron variant in China: a modeling study
Source: Infect Dis Poverty. 2023 Jan 16;12:1. doi: 10.1186/s40249-022-01049-w (PMC9841147; doi:10.1186/s40249-022-01049-w)
Supplement: Supplementary file 2 — Additional file 2: Supplements of the results. Fig. S3. The number of infections in 100 days for each strategy (P2). Fig. S4. The cumulative infection rates of 1000 simulations (P3). Fig. S5. The number of people quarantined in the hotel in 100 days for each strategy (P4). Fig. S6. The number of people quarantined in the shelter in 100 days for each strategy (P5). Fig. S7. The number of hospitalized cases in 100 days for each strategy (P6). Fig. S8. Cumulative infection rate, miss rate, and tests per positive of screening at different times (P7). Fig. S9. Cumulative and the maximum number of quarantine and hospitalized people of screening at different times (P7). Fig. S10. Cumulative infection rate, miss rate, tests per positive, the number of quarantining and hospitalization when ${{R}}_{0}$R0 = 2.5 (P8). Fig. S11. Cumulative infection rate, miss rate, tests per positive, the number of quarantining and hospitalization when ${{R}}_{0}$R0 = 5 (P9). Fig. S12. Cumulative infection rate, miss rate, tests per positive, the number of quarantining and hospitalization when $\alpha$α = 0.5 (P10). Fig. S13. Cumulative infection rate, miss rate, tests per positive, the number of quarantining and hospitalization when [file 40249_2022_1049_MOESM2_ESM.docx]

**Additional file 2**

**Contexts**

Fig. S3 The number of infections in 100 days for each strategy (P2)

Fig. S4 The cumulative infection rates of 1000 simulations (P3)

Fig. S5 The number of people quarantined in the hotel in 100 days for each strategy (P4)

Fig. S6 The number of people quarantined in the shelter in 100 days for each strategy (P5)

Fig. S7 The number of hospitalized cases in 100 days for each strategy (P6)

Fig. S8 Cumulative infection rate, miss rate, and tests per positive of screening at different times (P7)

Fig. S9 Cumulative and the maximum number of quarantine and hospitalized people of screening at different times (P7)

Fig. S10 Cumulative infection rate, miss rate, tests per positive, the number of quarantining and hospitalization when $\text{R}_{\text{0}}$=2$\text{.}$5 (P8)

Fig. S11 Cumulative infection rate, miss rate, tests per positive, the number of quarantining and hospitalization when $\text{R}_{\text{0}}$=5 (P9)

Fig. S12 Cumulative infection rate, miss rate, tests per positive, the number of quarantining and hospitalization when $\text{α}$=0.5 (P10)

Fig. S13 Cumulative infection rate, miss rate, tests per positive, the number of quarantining and hospitalization when $\text{α}$=0.7 (P11)

Fig. S14 Cumulative infection rate, miss rate, tests per positive, the number of quarantining and hospitalization when $\text{ds}$=0.7213475 (P12)

Fig. S15 Cumulative infection rate, miss rate, tests per positive, the number of quarantining and hospitalization when $\text{ds}$ =3.4760595 (P13)

Fig. S16 Cumulative infection rate, miss rate, tests per positive, the number of quarantining and hospitalization when $\text{p}_{\text{casual}}$=0.1 (P14)

Fig. S17 Cumulative infection rate, miss rate, tests per positive, the number of quarantining and hospitalization when $\text{p}_{\text{casual}}$=0.2 (P15)

Fig. S18 Cumulative infection rate, miss rate, tests per positive, the number of quarantining and hospitalization when $\text{p}_{\text{contact}}$=0.85 (P16)

Fig. S19 Cumulative infection rate, miss rate, tests per positive, the number of quarantining and hospitalization when $\text{p}_{\text{contact}}$=0.95 (P17)


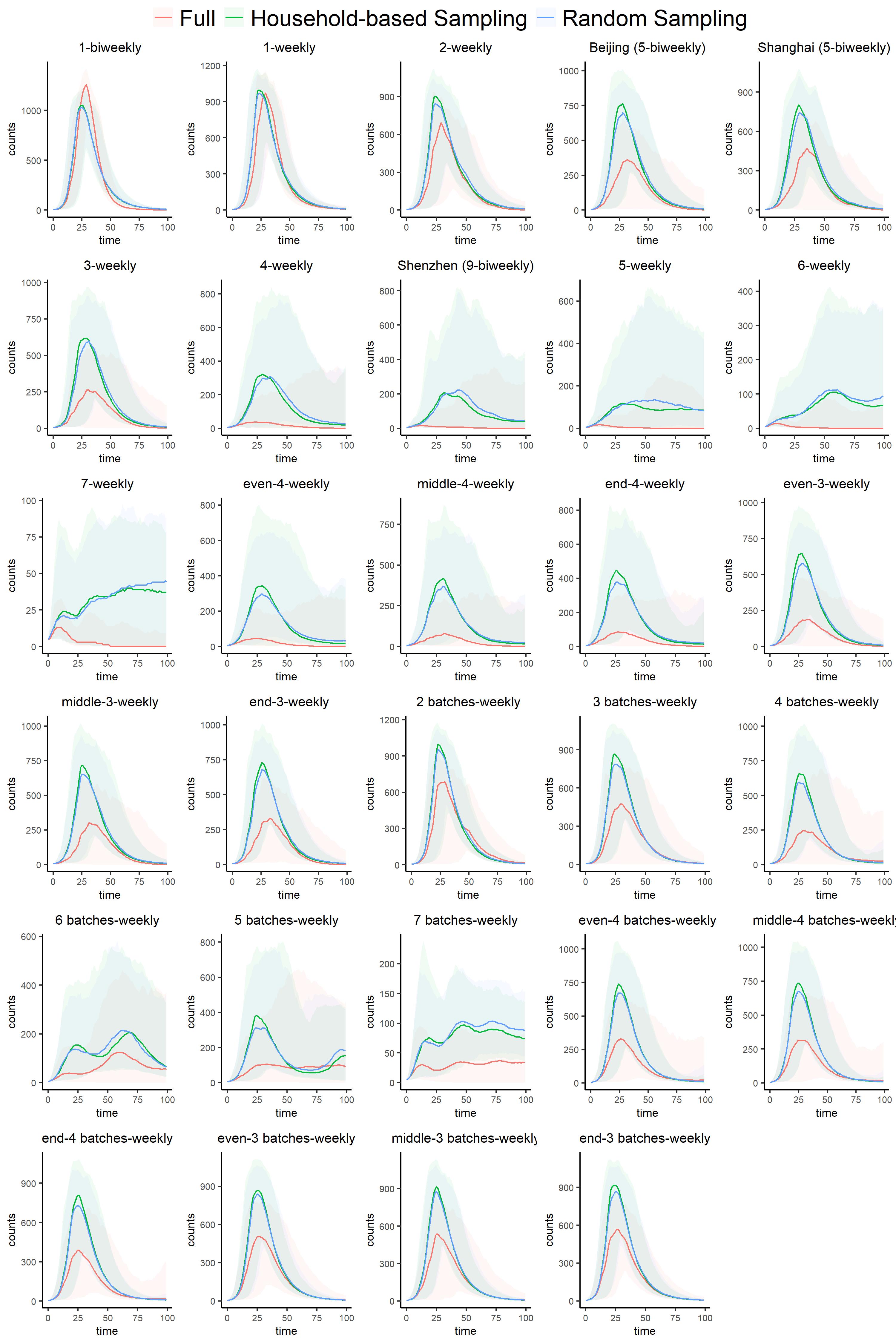


**Fig. S3 The number of infections in 100 days for each strategy**


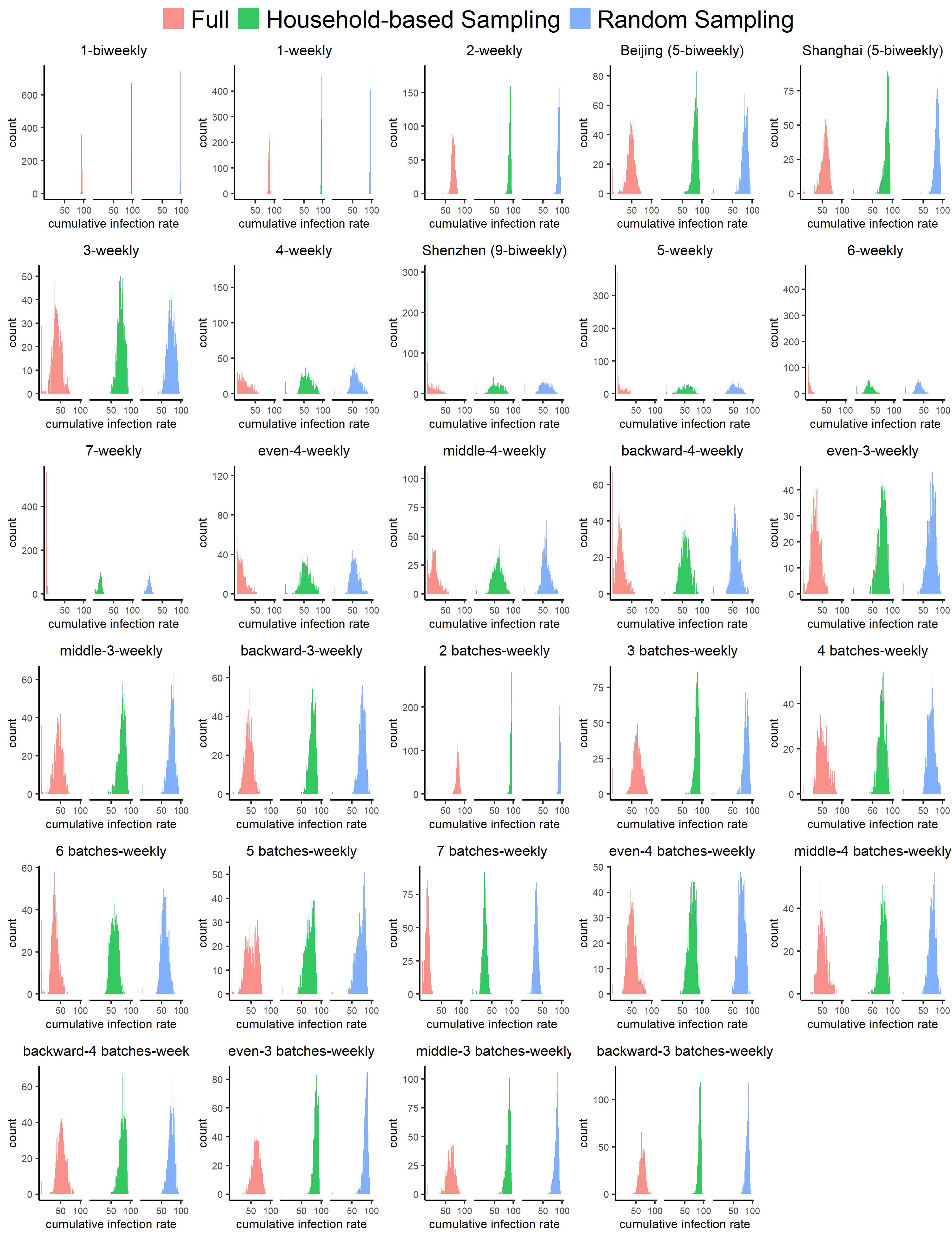


**Fig. S4 The cumulative infection rates of 1000 simulations**


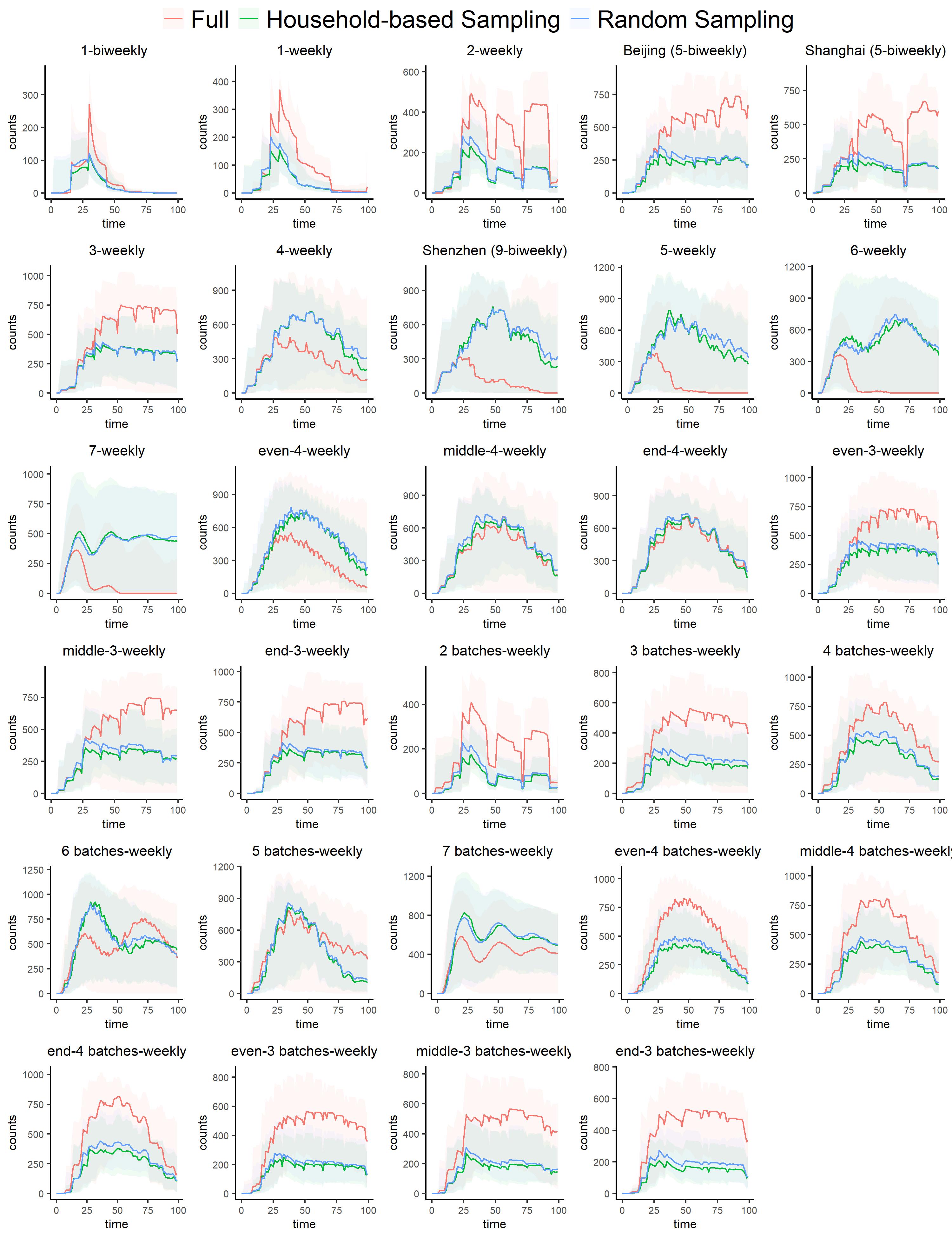


**Fig. S5 The number of people quarantined in the hotel in 100 days for strategies**


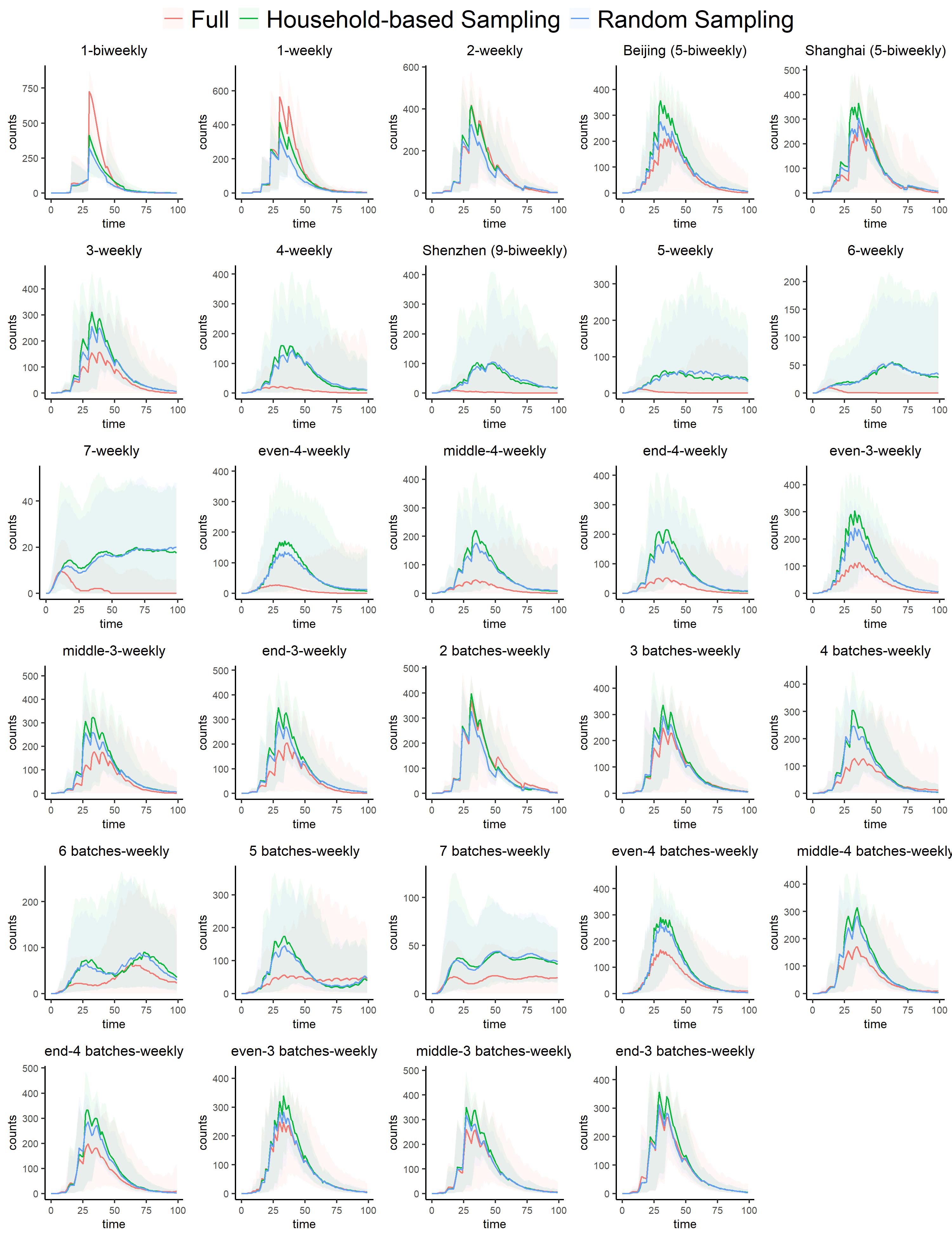


**Fig. S6 The number of people quarantined in the shelter in 100 days for strategies**


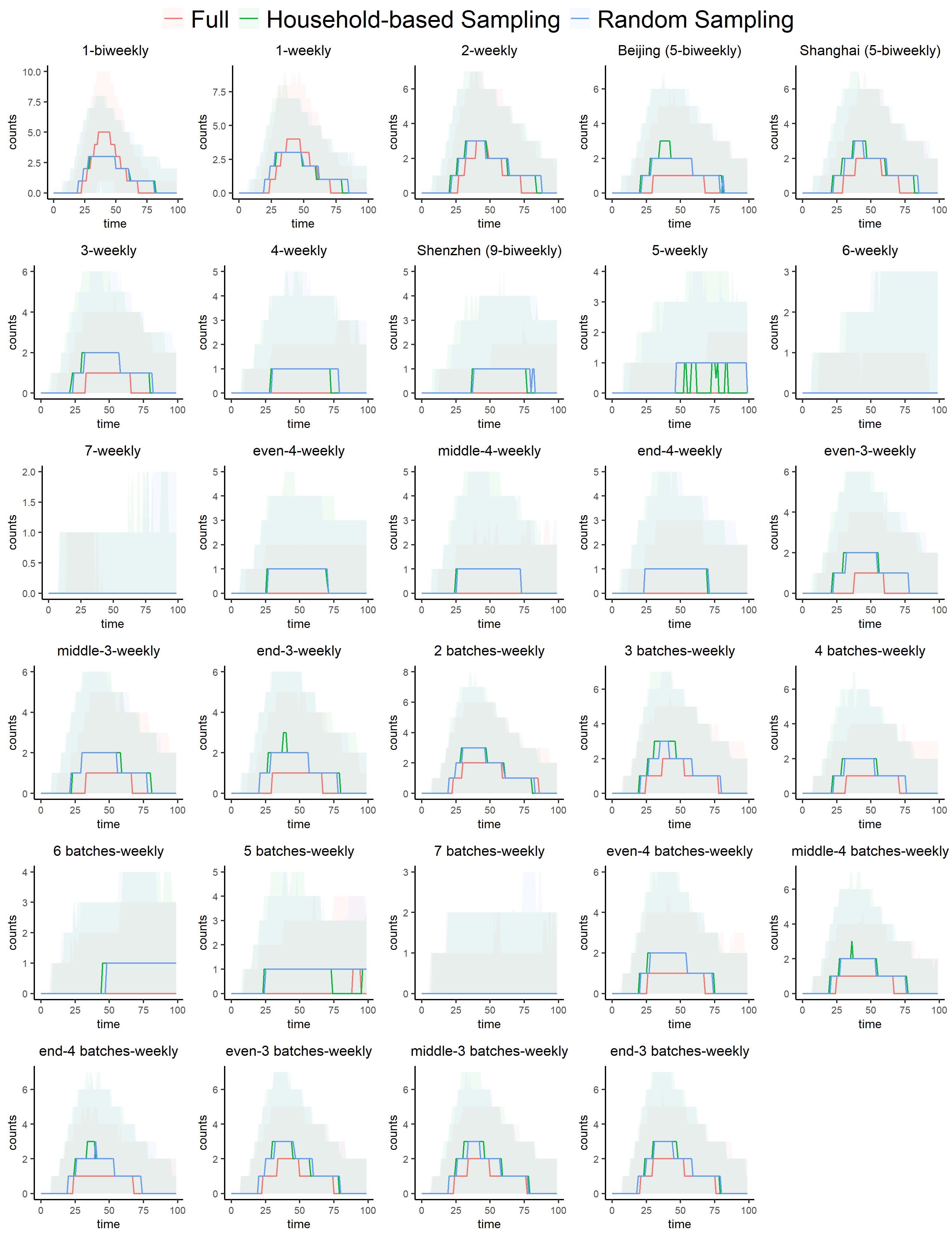


**Fig. S7 The number of hospitalized cases in 100 days for strategies**


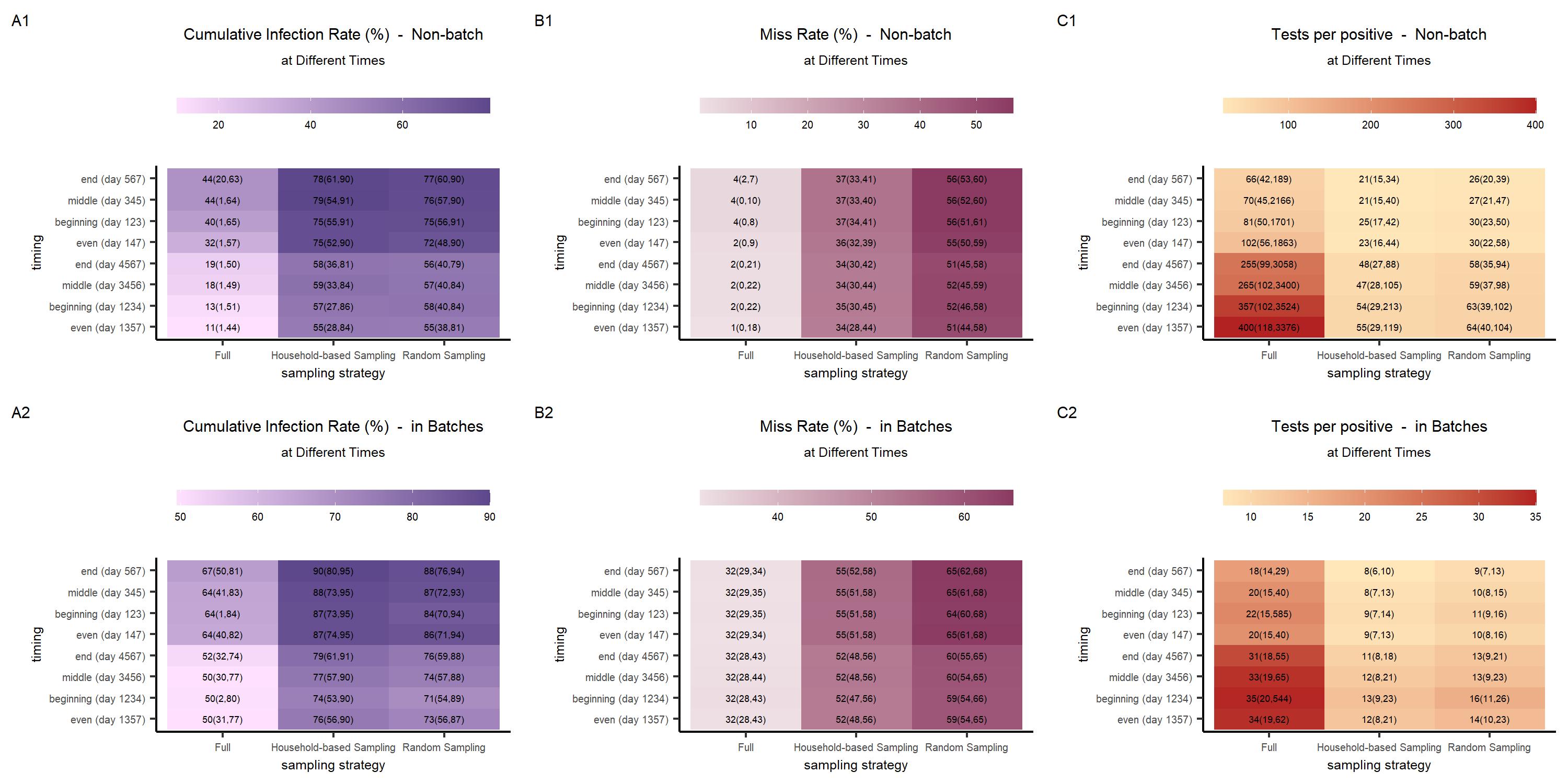


**Fig. S8 Cumulative infection rate, miss rate, and tests per positive of screening at different times**


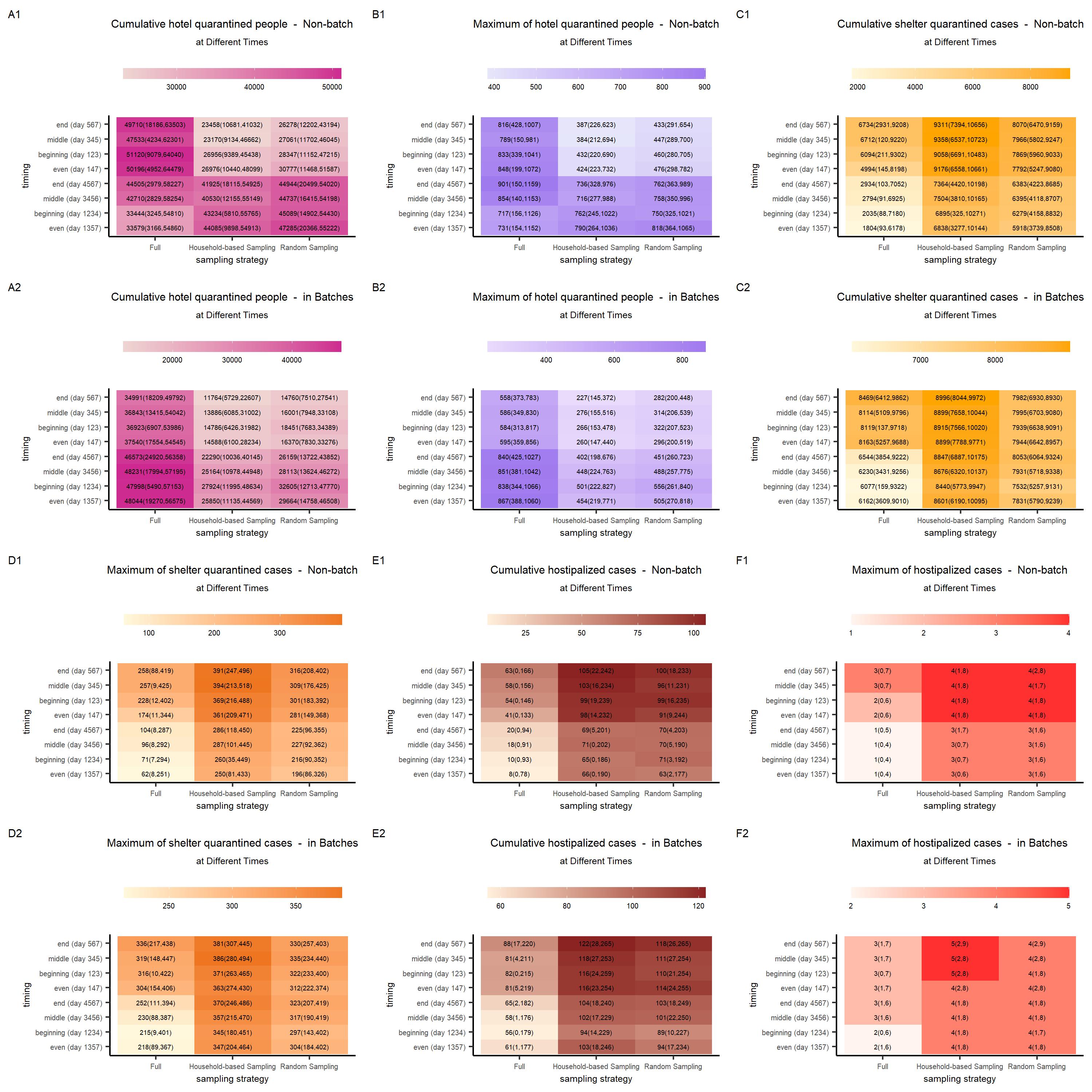


**Fig. S9 Cumulative and the maximum number of quarantine and hospitalized people of screening at different times**


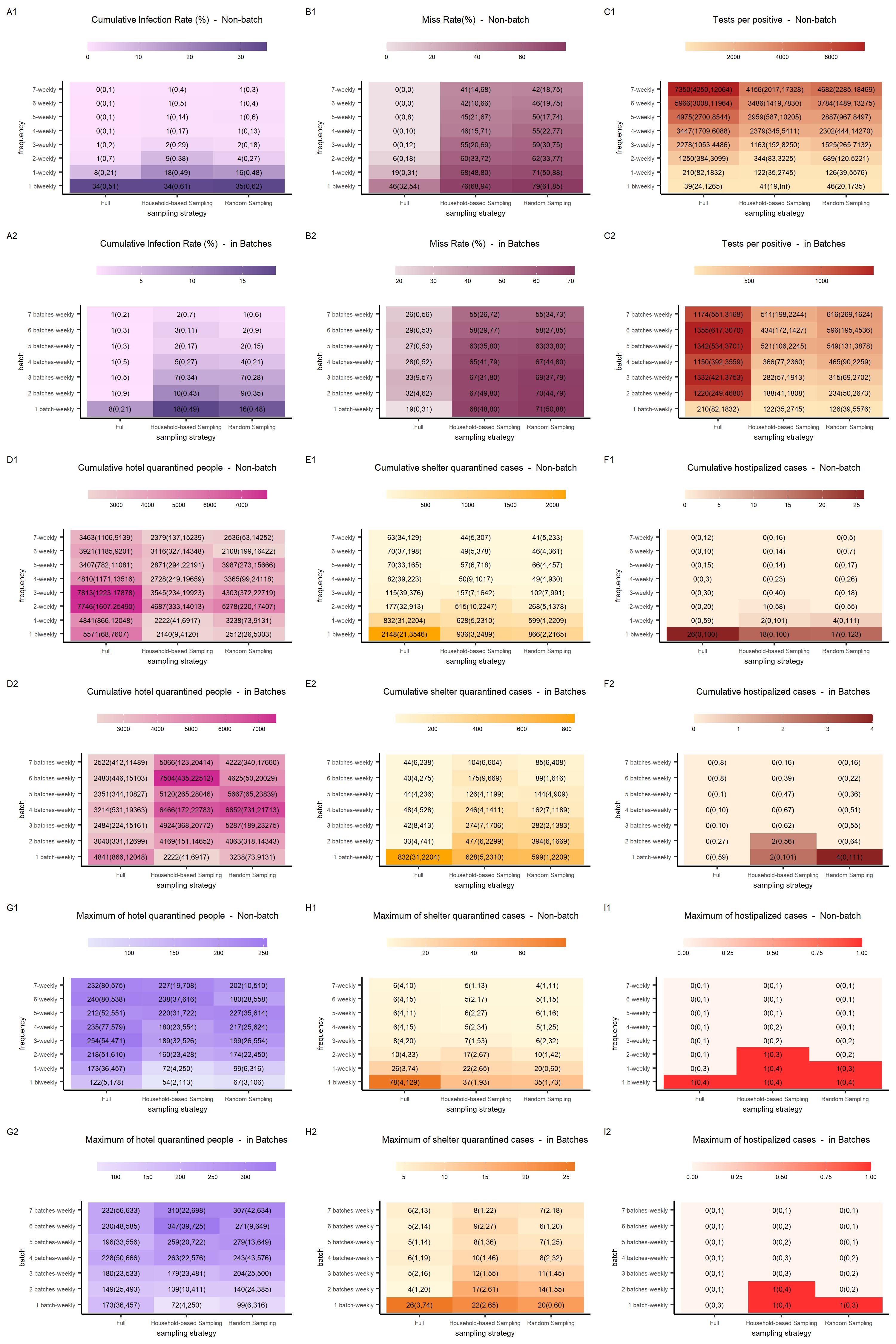


**Fig. S10** **Cumulative infection rate, miss rate, tests per positive, the number of quarantining and hospitalization when** $\text{R}_{\text{0}}$**=2**$\text{.}$**5**


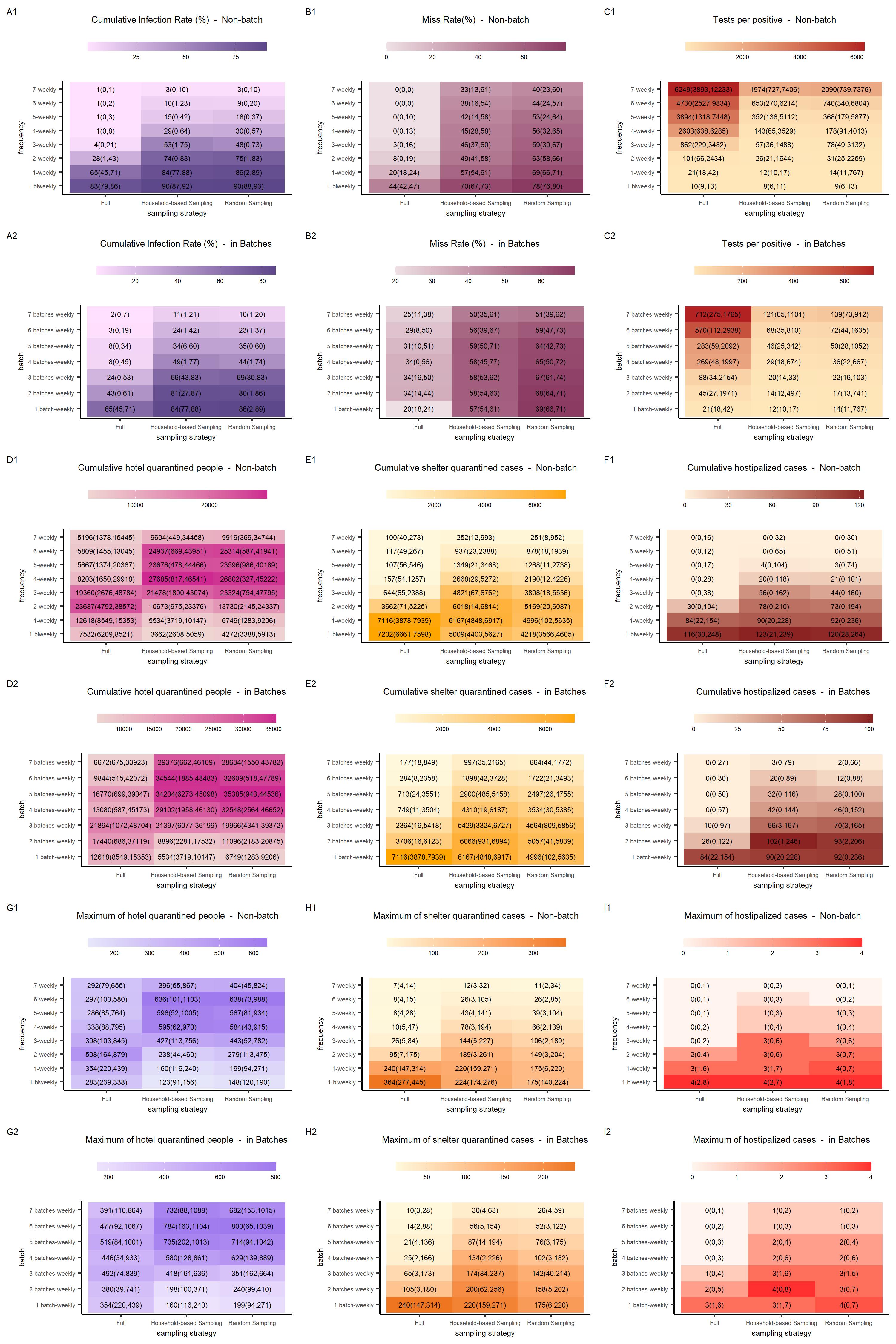


**Fig. S11 Cumulative infection rate, miss rate, tests per positive, the number of quarantining and hospitalization when** $\text{R}_{\text{0}}$**=5**


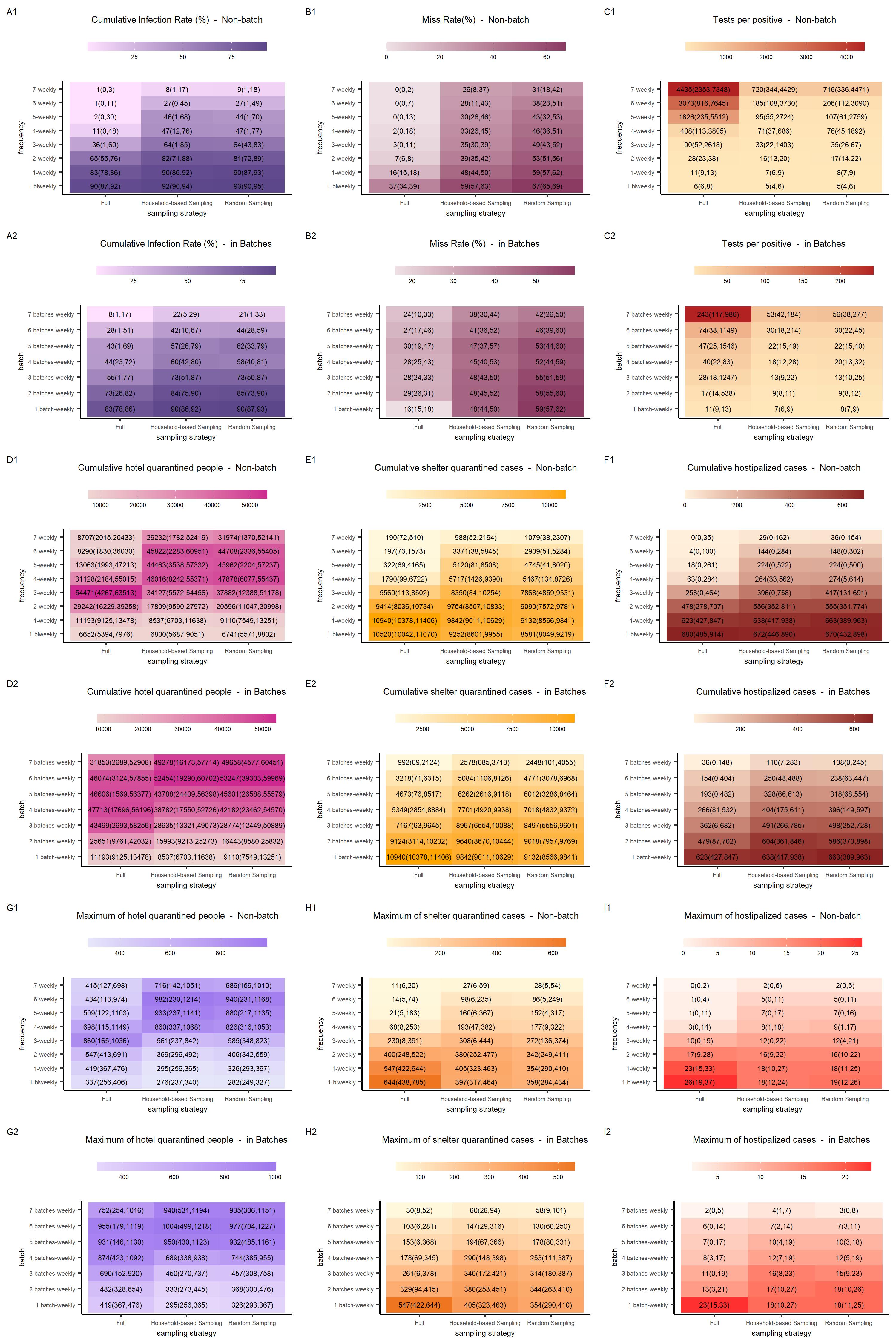


**Fig. S12 Cumulative infection rate, miss rate, tests per positive, the number of quarantining and hospitalization when** $\text{α}$**=0**$\text{.}$**5**


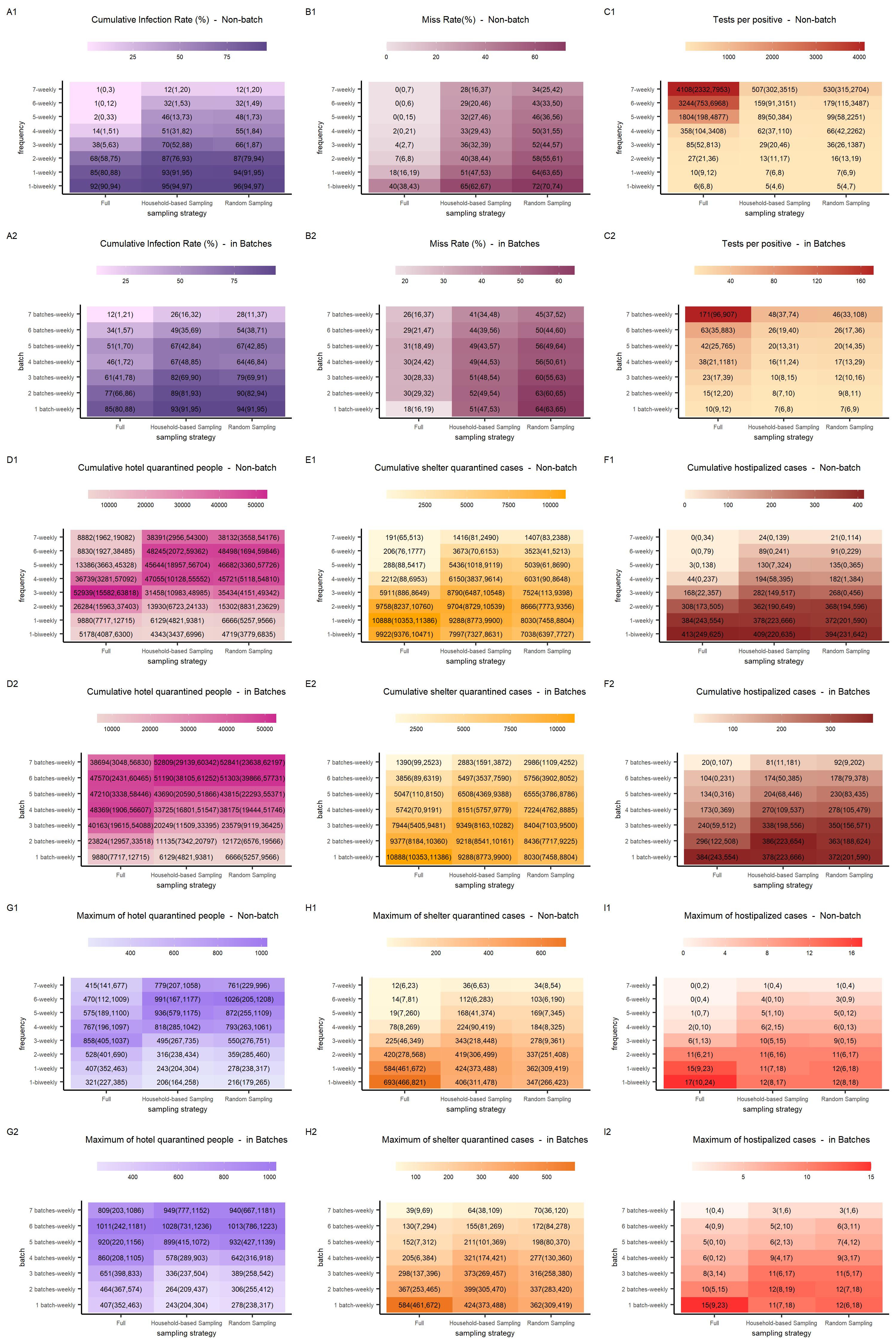


**Fig. S13 Cumulative infection rate, miss rate, tests per positive, the number of quarantining and hospitalization when** $\text{α}$**=0**$\text{.}$**7**


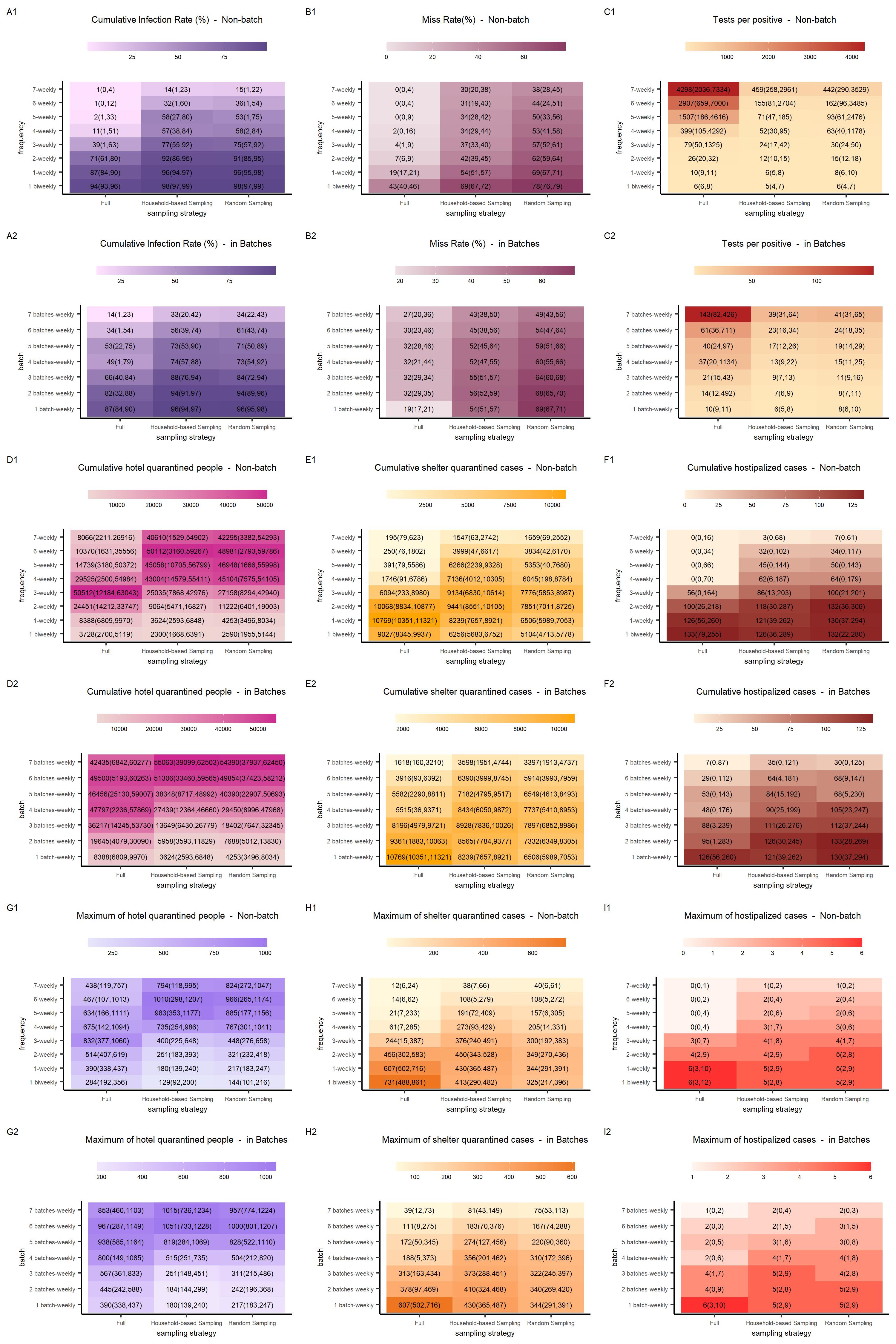


**Fig. S14 Cumulative infection rate, miss rate, tests per positive, the number of quarantining and hospitalization when** $\text{ds}$**=0**$\text{.}$**7213475**


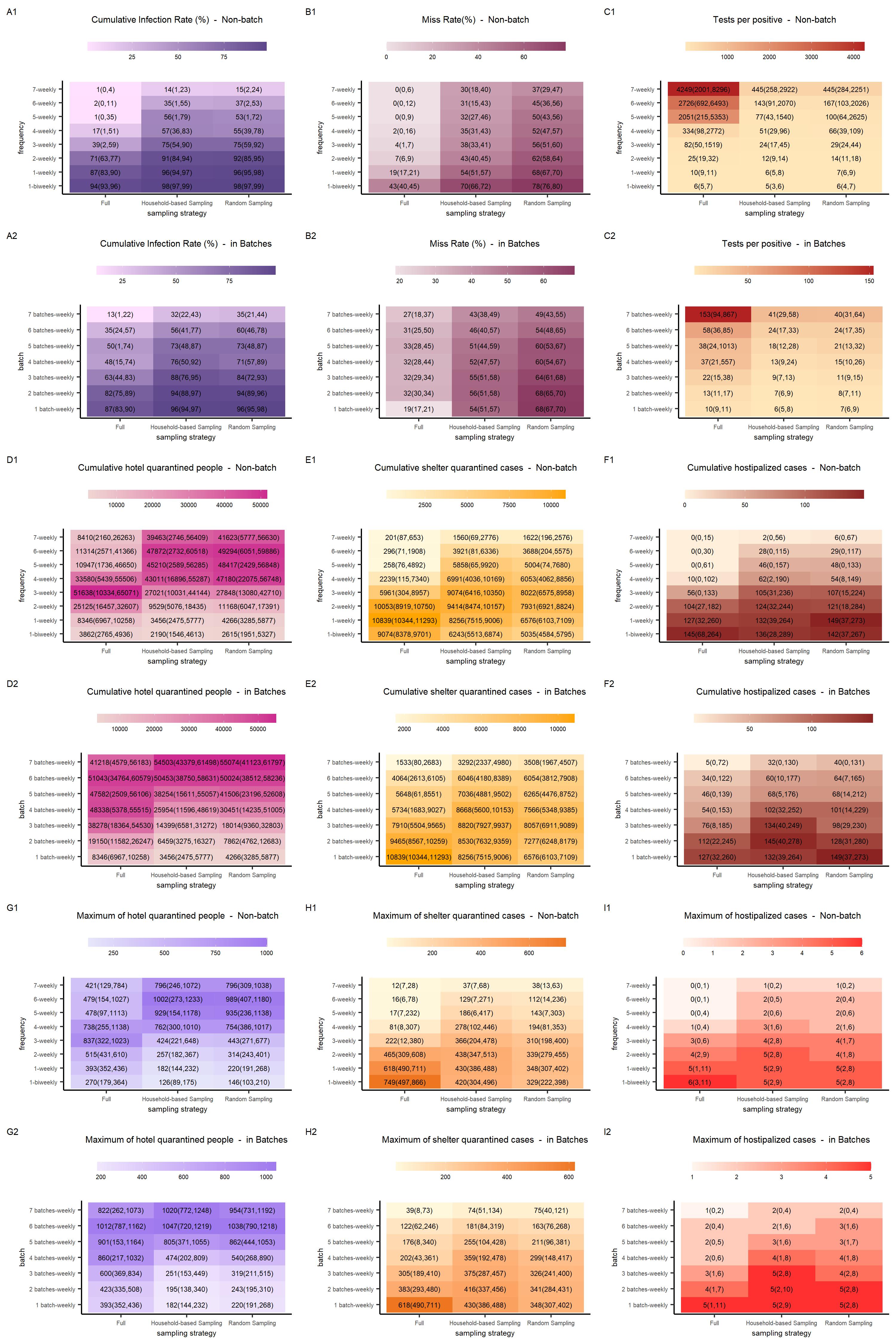


**Fig. S15 Cumulative infection rate, miss rate, tests per positive, the number of quarantining and hospitalization when** $\text{ds}$**=3**$\text{.}$**4760595**


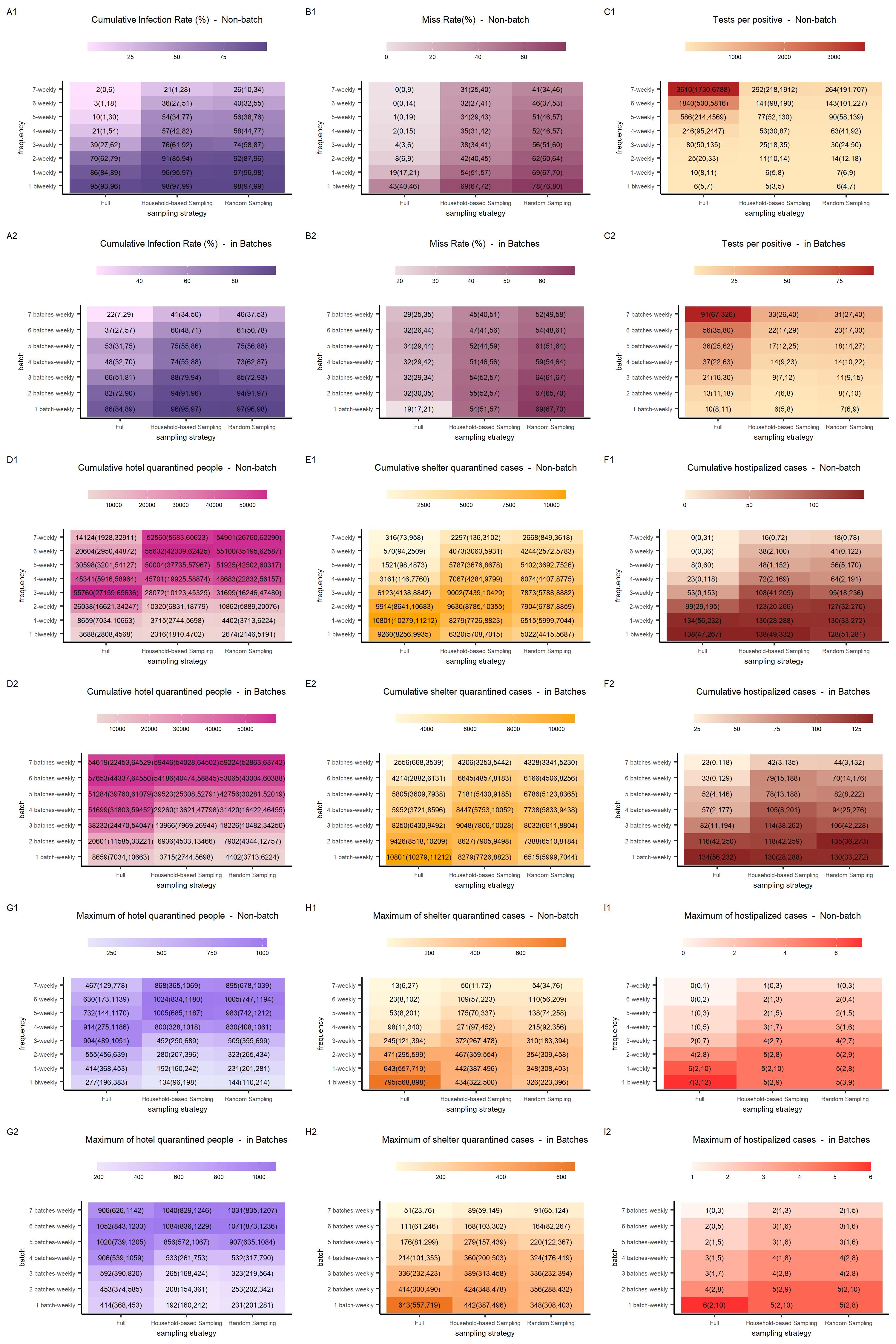


**Fig. S16 Cumulative infection rate, miss rate, tests per positive, the number of quarantining and hospitalization when** $\text{p}_{\text{casual}}$**=0**$\text{.}$**1**


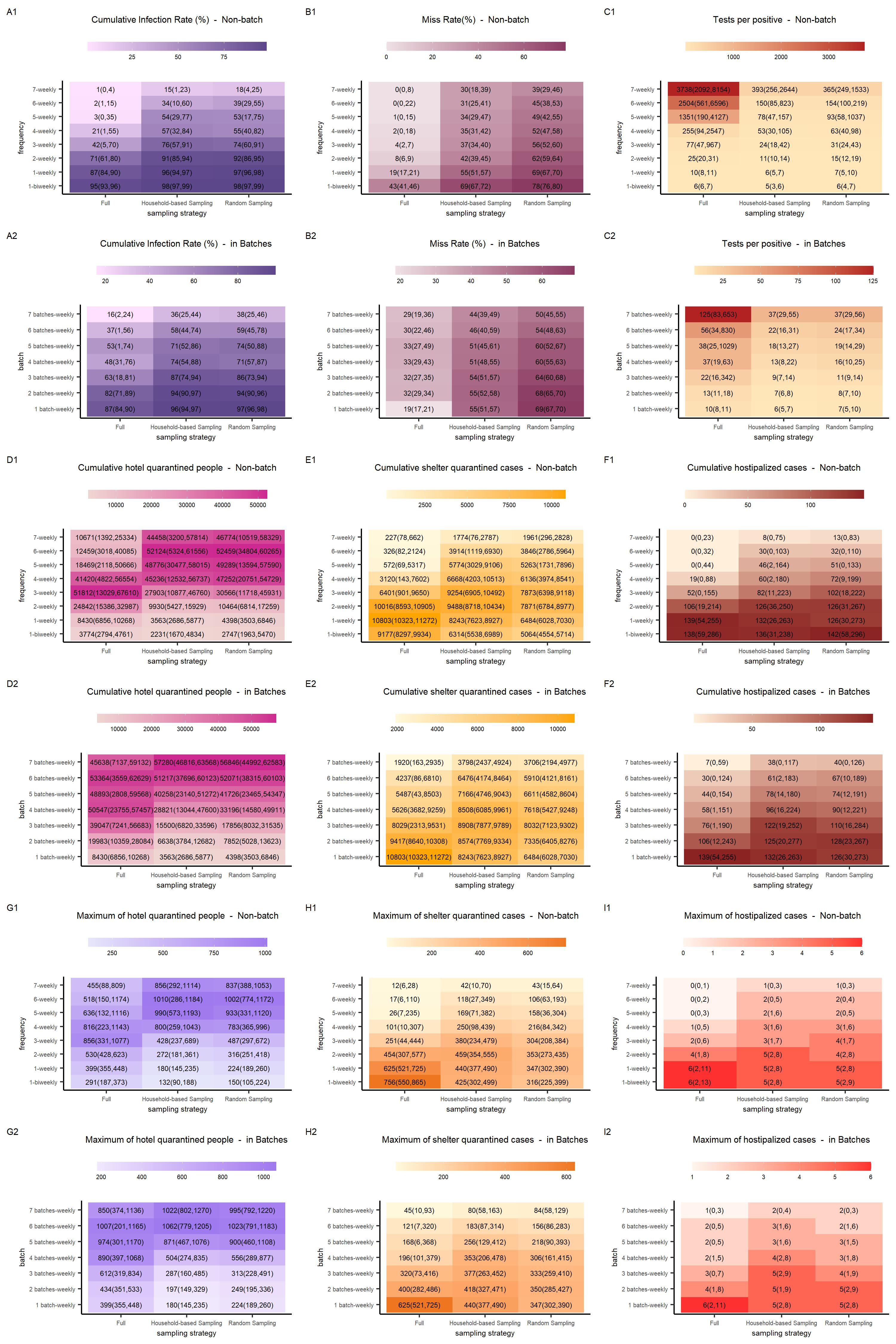


**Fig. S17 Cumulative infection rate, miss rate, tests per positive, the number of quarantining and hospitalization when** $\text{p}_{\text{casual}}$**=0**$\text{.}$**2**


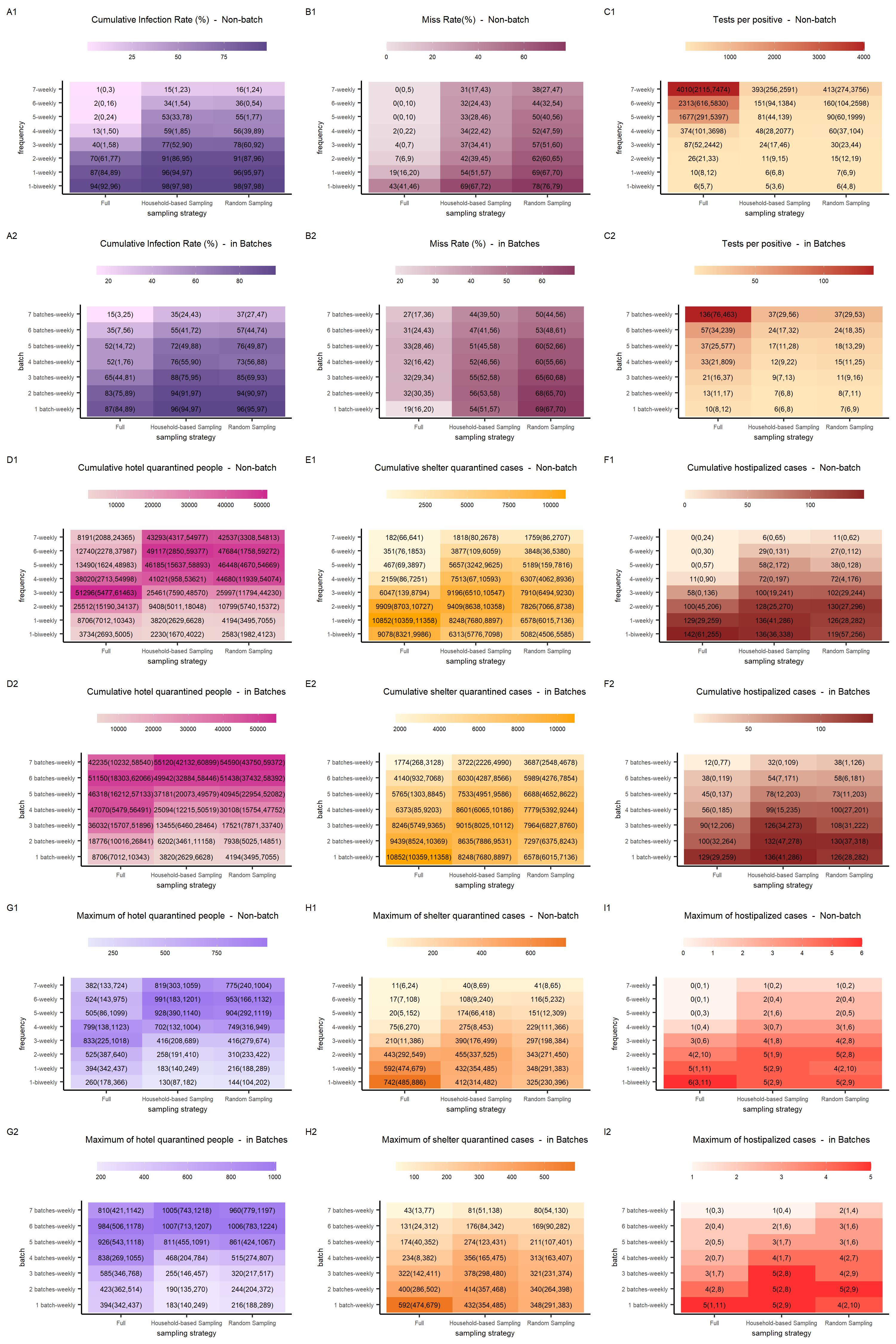


**Fig. S18 Cumulative infection rate, miss rate, tests per positive, the number of quarantining and hospitalization when** $\text{p}_{\text{contact}}$**=0**$\text{.}$**85**


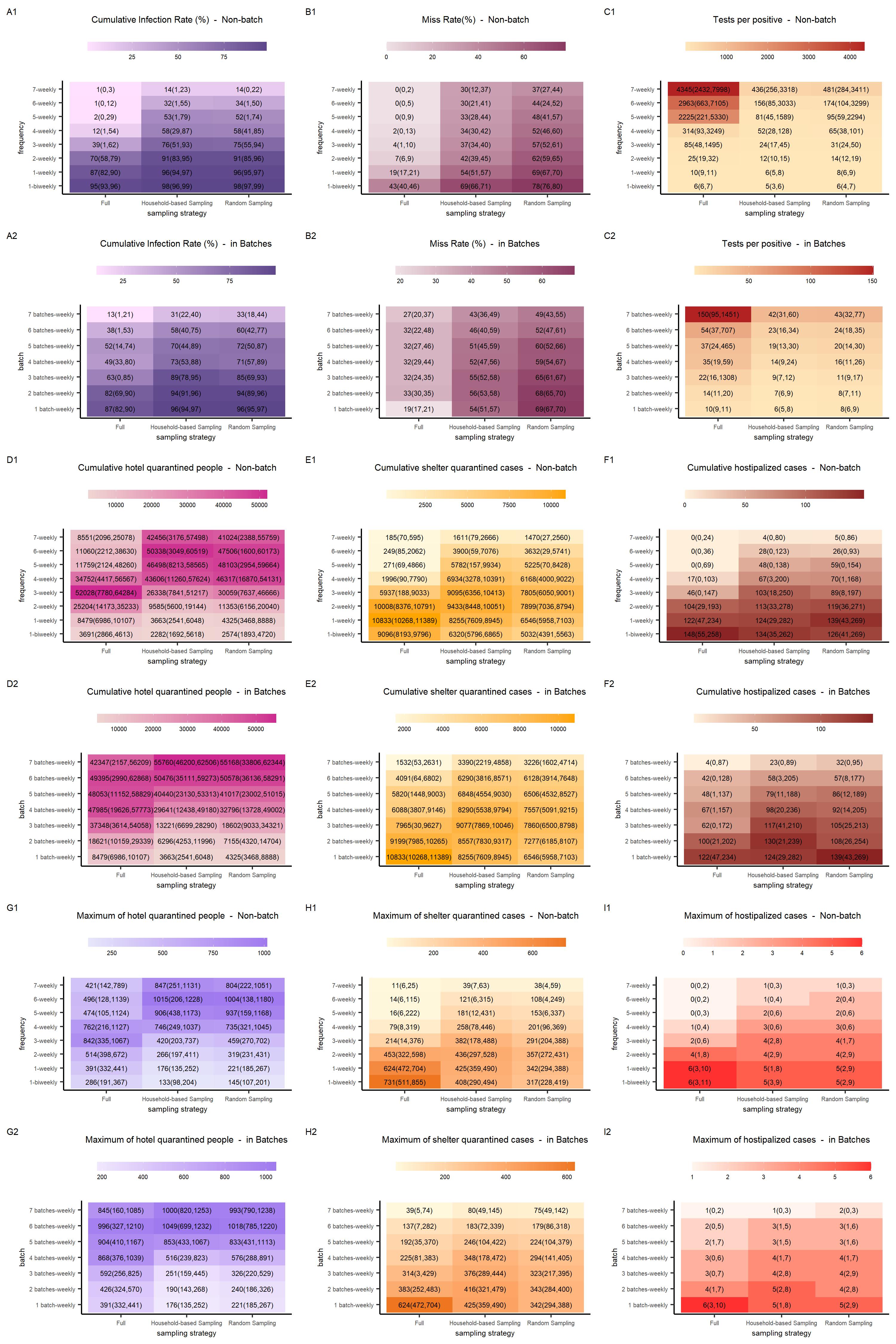


**Fig. S19 Cumulative infection rate, miss rate, tests per positive, the number of quarantining and hospitalization when** $\text{p}_{\text{contact}}$**=0**$\text{.}$**95**
